# Supplementary material for: Promoting teaching innovation of Chinese public-school teachers by team temporal leadership: The mediation of job autonomy and the moderation of work stress
Source: PLoS One. 2022 Jul 8;17(7):e0271195. doi: 10.1371/journal.pone.0271195 (PMC9269914; doi:10.1371/journal.pone.0271195)
Supplement: Attachment — Submitted filename: 2022_May9_Recommended edits for authors.pdf [file pone.0271195.s002.pdf]

<u>Recommended edits for authors:</u>

Line 24: delete “to”

Line 31: change “emphasize” to “emphasizing”

Line 47: add space between ”association” and “between”

Line 79: delete “JDR”

Line 98: use of “JD-R” makes sense here and subsequently

Line 159: re: Chen and Hu theory, is this a six- or a four-factor model since only four categories are provided?

Line 215: add “along with” before “decrease burnout and conflict”

Line 251: delete “of”

Line 252: change “Autonomy” to lower case “autonomy”

Line 253: same as line 252

Line 284: delete “leaderships”

Lines 284-285: recommend removing reference to Eagly et al. since “It is especially emphasized by laissez-faire leadership.” since this seems to be taken out of context to workplace autonomy

Line 289: need to clarify “the latter”, do you mean related to transformational leadership?

Line 340: change “People” to lower case “people”

Line 396: change “management” to “management field”

Line 396: change “targeting” to “target”

Line 40(: change “There were seven items” to “The seven items”

Lien 416: change “I” to “The”

Line 421: add space after “;”

Lines 419-424: the order presented should also be referenced in the Results section by starting with current section “4.2 Mean values, standard deviations, and correlation matrixes for each variable” first and then move section “4.1 Common method bias” after the new section 4.1 as the new section 4.2

Figures should include the footnotes for the different levels of significance
