# Supplementary material for: Promoting teaching innovation of Chinese public-school teachers by team temporal leadership: The mediation of job autonomy and the moderation of work stress
Source: PLoS One. 2022 Jul 8;17(7):e0271195. doi: 10.1371/journal.pone.0271195 (PMC9269914; doi:10.1371/journal.pone.0271195)

PLOS ONE

Promoting Teaching Innovation by Team Temporal Leadership: The Mediation of Job Autonomy and the modulation of Work Stress

--Manuscript Draft--

Manuscript Number:	PONE-D-22-11897
Article Type:	Research Article
Full Title:	Promoting Teaching Innovation by Team Temporal Leadership: The Mediation of Job Autonomy and the modulation of Work Stress
Short Title:	Team Temporal Leadership and Teaching Innovation
Corresponding Author:	Guiqin Zhu, Ph.D<br>Chongqing Normal University - University Town Campus: Chongqing Normal University<br>Chongqing, Chongqing CHINA
Keywords:	temporal leadership; innovation; teachers; job autonomy; Stress; teaching effectiveness
Abstract:	This study examines the impact of team temporal leadership, the behaviors of supervisors regarding to scheduling, allocating time resources, and coordinating team members, on teachers' innovative behavior. Questionnaire surveys on 2021 elementary and secondary school teachers show that team temporal leadership exerts a significant positive direct effect on teaching innovation and the effect can be facilitated through the mediation of job autonomy. Moreover, both the direct effect and the second-leg of the mediation effect are modulated by work stress. These suggest that teaching innovation can benefit from leaders' appropriate scheduling and synchronization of time resources, while emphasize the roles of job autonomy and work stress during this time-based team management.
Order of Authors:	Kai Li
	Guiqin Zhu, Ph.D
Additional Information:	
**Question**	**Response**
**Financial Disclosure**<br><br>Enter a financial disclosure statement that describes the sources of funding for the work included in this submission. Review the submission guidelines for detailed requirements. View published research articles from *PLOS ONE* for specific examples.<br><br>This statement is required for submission and **will appear in the published article** if the submission is accepted. Please make sure it is accurate.	Recipient: Zhu, G<br>Grant Number: BHA18019<br>National Education Sciences Planning Project, China

Powered by Editorial Manager® and ProduXion Manager® from Aries Systems Corporation

**Unfunded studies**<br>Enter: *The author(s) received no specific funding for this work.*<br><br>**Funded studies**<br>Enter a statement with the following details:<br>• Initials of the authors who received each award<br>• Grant numbers awarded to each author<br>• The full name of each funder<br>• URL of each funder website<br>• Did the sponsors or funders play any role in the study design, data collection and analysis, decision to publish, or preparation of the manuscript?<br>• **NO** - Include this sentence at the end of your statement: *The funders had no role in study design, data collection and analysis, decision to publish, or preparation of the manuscript.*<br>• **YES** - Specify the role(s) played.<br><br>* typeset	
**Competing Interests**<br><br>Use the instructions below to enter a competing interest statement for this submission. On behalf of all authors, disclose any competing interests that could be perceived to bias this work—acknowledging all financial support and any other relevant financial or non-financial competing interests.<br><br>This statement is required for submission and **will appear in the published article** if the submission is accepted. Please make sure it is accurate and that any funding sources listed in your Funding Information later in the submission form are also declared in your Financial Disclosure statement.<br><br>View published research articles from *PLOS ONE* for specific examples.	No conflicts of interest are declared. All authors have approved the final article.

**NO authors have competing interests**<br><br>Enter: *The authors have declared that no competing interests exist.*<br><br>**Authors with competing interests**<br><br>Enter competing interest details beginning with this statement:<br><br>*I have read the journal's policy and the authors of this manuscript have the following competing interests: [insert competing interests here]*<br><br>* typeset	
**Ethics Statement**<br><br>Enter an ethics statement for this submission. This statement is required if the study involved:<br><br>• Human participants<br>• Human specimens or tissue<br>• Vertebrate animals or cephalopods<br>• Vertebrate embryos or tissues<br>• Field research<br><br>Write "N/A" if the submission does not require an ethics statement.<br><br>General guidance is provided below. Consult the submission guidelines for detailed instructions. **Make sure that all information entered here is included in the Methods section of the manuscript.**	The study was approved by the Ethic Committee of Xinyang Normal University (No. XYEC-2021-009), all the teachers gave written consents for participation.

Powered by Editorial Manager® and ProduXion Manager® from Aries Systems Corporation

**Format for specific study types**<br><br>**Human Subject Research (involving human participants and/or tissue)**<br>• Give the name of the institutional review board or ethics committee that approved the study<br>• Include the approval number and/or a statement indicating approval of this research<br>• Indicate the form of consent obtained (written/oral) or the reason that consent was not obtained (e.g. the data were analyzed anonymously)<br><br>**Animal Research (involving vertebrate animals, embryos or tissues)**<br>• Provide the name of the Institutional Animal Care and Use Committee (IACUC) or other relevant ethics board that reviewed the study protocol, and indicate whether they approved this research or granted a formal waiver of ethical approval<br>• Include an approval number if one was obtained<br>• If the study involved *non-human primates*, add *additional details* about animal welfare and steps taken to ameliorate suffering<br>• If anesthesia, euthanasia, or any kind of animal sacrifice is part of the study, include briefly which substances and/or methods were applied<br><br>**Field Research**<br><br>Include the following details if this study involves the collection of plant, animal, or other materials from a natural setting:<br>• Field permit number<br>• Name of the institution or relevant body that granted permission	
**Data Availability**<br><br>Authors are required to make all data underlying the findings described fully available, without restriction, and from the time of publication. PLOS allows rare exceptions to address legal and ethical concerns. See the PLOS Data Policy and FAQ for detailed information.	Yes - all data are fully available without restriction

A Data Availability Statement describing where the data can be found is required at submission. Your answers to this question constitute the Data Availability Statement and **will be published in the article**, if accepted.<br><br>**Important:** Stating 'data available on request from the author' is not sufficient. If your data are only available upon request, select 'No' for the first question and explain your exceptional situation in the text box.<br><br>Do the authors confirm that all data underlying the findings described in their manuscript are fully available without restriction?	
**Describe where the data may be found in full sentences. If you are copying our sample text, replace any instances of XXX with the appropriate details.**<br><br>• If the data are **held or will be held in a public repository**, include URLs, accession numbers or DOIs. If this information will only be available after acceptance, indicate this by ticking the box below. For example: *All XXX files are available from the XXX database (accession number(s) XXX, XXX.*).<br>• If the data are all contained **within the manuscript and/or Supporting Information files**, enter the following: *All relevant data are within the manuscript and its Supporting Information files.*<br>• If neither of these applies but you are able to provide **details of access elsewhere**, with or without limitations, please do so. For example:<br><br>*Data cannot be shared publicly because of [XXX]. Data are available from the XXX Institutional Data Access / Ethics Committee (contact via XXX) for researchers who meet the criteria for access to confidential data.*<br><br>*The data underlying the results presented in the study are available from (include the name of the third party*	Data for this article can be accessed via https://osf.io/89xnh/.

Powered by Editorial Manager® and ProduXion Manager® from Aries Systems Corporation

and contact information or URL).<br>• This text is appropriate if the data are owned by a third party and authors do not have permission to share the data.<br><br>* typeset	
Additional data availability information:	Tick here if the URLs/accession numbers/DOIs will be available only after acceptance of the manuscript for publication so that we can ensure their inclusion before publication.

Powered by Editorial Manager® and ProduXion Manager® from Aries Systems Corporation

Manuscript

Click here to access/download;Manuscript;plos20220422_manuscript_witha

Promoting Teaching Innovation by Team Temporal Leadership: The Mediation of Job Autonomy and the modulation of Work Stress

Kai Li[1], Guiqin Zhu*[2]

1 School of Management, Xi'an University of Architecture and Technology, Xi'an, Shaanxi, 710055, China

2 School of Education, Chongqing Normal University, 401331, Chongqing, China

*** Corresponding authors:**

Guiqin Zhu, Ph.D., School of Education, Chongqing Normal University, Chongqing, 401331, China

Email: qin7.7@126.com

Promoting Teaching Innovation by Team Temporal Leadership: The Mediation of Job Autonomy and the modulation of Work Stress

Abstract

This study examines the impact of team temporal leadership, the behaviors of supervisors regarding to scheduling, allocating time resources, and coordinating team members, on teachers' innovative behavior. Questionnaire surveys on 2021 elementary and secondary school teachers show that team temporal leadership exerts a significant positive direct effect on teaching innovation and the effect can be facilitated through the mediation of job autonomy. Moreover, both the direct effect and the second-leg of the mediation effect are modulated by work stress. These suggest that teaching innovation can benefit from leaders' appropriate scheduling and synchronization of time resources, while emphasize the roles of job autonomy and work stress during this time-based team management.

Keywords: temporal leadership, innovation, teachers, job autonomy, stress, teaching effectiveness

1. Introduction

Innovation, the process “leading to creative learning by generating and implementing new ideas, methods, tools, and content” (Zhu & Di, 2014; Zhu et al., 2013), is at the heart of contemporary education. It promotes teacher effectiveness and stimulates children's motivation, learning, and curiosity (Rinkevich, 2011). As teachers are at the forefront of developing and implementing new ideas, their innovative behavior is critical for the effectiveness and sustainability of school organizations (Thurlings et al., 2015). While innovative behavior can be self-initiated at the individual level, innovation in educational organizations is largely a collective, complex knowledge-based task in the orchestration between the teachers and leaders (Nguyen et al., 2021; Vangrieken et al., 2017). Existing models and empirical studies have established close association between leadership and innovative work performance (Hammond et al., 2011; Soleas, 2020) and innovation in teaching in particular is contingent on leadership behaviors (Keane et al., 2020; Thurlings et al., 2015, Marks & Printy, 2003; Rashid & Halim, 2015).

Innovation requires time. However, teachers nowadays face stressors on many fronts: increasing family responsibilities, task overload, student heterogeneity, evolving pedagogy and curriculum reforms, information explosion, just to name a few (Cai & Tang, 2021; Nguyen et al., 2021). Additionally, when teachers collaborate to improve instructional ideas and technologies, there arises the problem of temporal diversity (Mohammed & Nadkarni, 2011) due to individual differences in ability, time urgency, time-based orientations, and pacing. Time scarcity and temporal diversity pose critical challenges to teachers' innovative behaviors. Therefore, appropriate leadership from school supervisors are required to help teachers restructure time resources, synchronize

rhythms, and avoid time-based conflicts.

In the last 20 years, the concept of temporal leadership (Mohammed & Nadkarni, 2011) has received increasing attention from scholars and practitioners amid intense calls for integrating time-related issues into models of leadership (Alipour et al., 2017; Ancona et al., 2001; Foti et al., 2017; Halbesleben et al., 2003). Temporal leadership refers to a unique module of leadership behaviors related to scheduling, allocating time resources, and coordinating team members in work rhythm, and helps the team achieve its goals by optimizing time resources (Mohammed & Nadkarni, 2011). This concept is considered a positive construct for organizational and employee performance. Importantly, in corporate and business settings, team supervisors' temporal leadership is positively associated with employees' innovative behavior (Halbesleben et al., 2003; Liu et al., 2021; Xiao et al., 2020; Zhang et al., 2021) and creativity (Gevers & Demerouti, 2013). It promotes innovation readiness (Chen & Liu, 2020), facilitates innovation, corporate venturing, and strategic renewal activities (Chen & Nadkarni, 2017). However, to the best of our knowledge, no report has yet been published on the impact of temporal leadership on teaching innovation.

Even if it can be observed that temporal leadership promotes teachers' innovative behaviors, other important questions arise such as: How is this realized through organizational processes and what are the boundary conditions? Drawing on existing evidence, particularly the work demands and resources (JD-R) theory (Bakker & Demerouti, 2014; Demerouti et al., 2001; Karasek, 1979), *we propose that temporal leadership positively affects teachers' innovative work behaviors and that its effect can be mediated by work resources such as work autonomy and modulated by work demands, including work stress.* Work autonomy reflects the amount of freedom people have to decide what to do in their jobs and how to do it (Karasek, 1979), and it is a

precondition for innovative behavior in the literature (Criscuolo et al., 2014). Stress is pervasive in the workplace and fluctuates with time and circumstances. It significantly affects job performance and innovative behavior (Daniel et al., 2020).

To test the hypothesis, we conducted questionnaire surveys of a representative sample of elementary and secondary school teachers (n = 2017) and analyzed the data according to the well-established conditional process model (Hayes & Rockwood, 2020). The questions of interests are t~~hat:~~ 1) ~~if~~ temporal leadership of principals positively predict~~ive~~ ~~of~~ innovative teacher behaviors; 2) ~~if~~ the effect of temporal leadership on teaching innovation could be mediated by job autonomy; and 3) ~~whether~~ the effect of temporal leadership on teaching innovation is modulated by work stress.

2 Theoretical Background and Hypothesis

2.1 The Job Demands-resources Theory

As one of the most widely cited theories in the field of organizational behavior, the Job demands and resources (JD-R) theory (Bakker & Demerouti, 2014, 2017; Demerouti et al., 2001) identifies two main categories of work environment that influence work engagement, performance, and well-being: Job demands and job resources. The former refers to "those physical, social, or organizational aspects of work that require sustained physical or mental effort and are therefore associated with certain physiological or psychological costs" (Bakker & Demerouti, 2014). The latter refers to "those physical, mental, social, or organizational aspects of work that are functional in (ⅰ) achieving work goals, (ⅱ) reducing work demands and associated costs, and (ⅲ) promoting personal growth, learning, and development" (Bakker & Demerouti, 2014). Typical examples of job demands are work pressure, emotional demands, and changing

tasks, and typical examples of job resources are organizational support, job autonomy, and time control. Job demands trigger a process of health impairment that impacts costs and leads to exhaustion and negative health problems, whereas job resources trigger a motivational process that is positive for work engagement and performance (Bakker & Demerouti, 2014). Whereas these two sets of factors set different processes in motion, they also work together to influence organizational outcomes: Work demands can modulate the impact of work resources on motivation and engagement. Among a number of factors, autonomy at work and work stress are two important variables in the JD-R theory. Work stress is also considered as the most important work demand and work autonomy as the most important work resource in the influential demand-control model (Karasek, 1979).

The JD-R theory has been well supported by empirical studies and modeling (see comprehensive reviews: (Bakker & Demerouti, 2007, 2014, 2017)). Levels of work resources have been found to be related to work engagement (Bakker et al., 2007), project success (Lattrich & Buettgen, 2020), and intention to leave work (Schaufeli & Bakker, 2004). High job demands predict burnout, health problems, and low well-being (Schaufeli & Bakker, 2004). There is also empirical evidence of the interaction between job demands and job resources (Bakker et al., 2007). The JD-R theory has been widely applied in recent decades in practices such as the JD-R monitor, organizational assessment, work design interventions, and personal resource interventions (Bakker & Demerouti, 2017). Critically, JD-R theory is also validated in innovative behaviors in organizations, workplaces, and educational institutions (Cao et al., 2020).

2.2 The team temporal leadership of school principals may affect teacher innovative work behavior

2.2.1 Team temporal leadership

The team temporal leadership concerns a unique dimension of leadership behavior that addresses time management in team activities (Ancona et al., 2001; Mohammed & Nadkarni, 2011; Myer & Mohammed, 2012), such as scheduling, time synchronization, and time resource allocation. As a multidimensional construct, temporal leadership includes two core components: task-oriented and relationship-oriented temporal leadership (Myer & Mohammed, 2012). The task-oriented dimension refers to leaders' behaviors that help structure, coordinate, and manage the pace of task completion, including reminding members of deadlines, scheduling time for contingencies and problems, and synchronizing members to get work done on time (Mohammed & Nadkarni, 2011; Myer & Mohammed, 2012). The relational dimension describes the behaviors of leaders that support members in managing time in the team, such as consulting with team members before making a decision about the schedule, dealing with time desynchronization among members, and praising members' performance on schedule (Myer & Mohammed, 2012). Previous studies have found it cushions the blow of temporal diversity on team performance (Najam et al., 2018; Mohammed & Nadkarni, 2011), and reduces time-based conflict when people with different styles of pace or time perspective work together (Najam et al., 2018). It promotes employee followership (Yuan & Lo, 2018), competence (Yuan & Lo, 2018), and professional engagement (Mudannayake et al., 2016).

2.2.2 Teaching Innovation

Some researchers conceptualize teaching innovation as "teachers' receptivity, openness, and willingness to embrace change" (Goldsmith, 1986; Nguyen et al., 2021), which acts "as a critical factor in teachers' innovative behavior and a prerequisite for innovation and change." Blândul (2015) identifies three main indicators of teaching innovation: information content, teaching materials, and teaching/assessment methods. In recent studies from East Asia, Chen and Hu (2008) propose a six-factor model of "creative teaching performance," including "new ideas," "new schemes," "updated materials," and "new assessment tools." Zhu et al. (2013) propose four core competencies, including "learning competence, pedagogical competence, social competence, and technological competence," and summarize three typical trends for innovative teaching and learning: "integrated use of information and communication technologies, the adoption of student-centered learning, and the use of collaborative learning approaches" (Zhu & Engels, 2014). Hung and Li (2017) advocate that "the content of innovative teaching can be categorized into five dimensions, namely idea thinking, curriculum content, teaching materials, teaching methods, and multifaceted assessment." This is in line with Hou's (2018) definition of teaching innovation, which proposes five similar dimensions of teaching innovation: " thinking innovation, content innovation, method innovation, resource innovation, and assessment innovation."

Although these definitions vary to some degree, they agree on some dimensions that are generally accepted. *Teaching innovation is generally understood to be the innovative behaviors of teachers in their workplace, encompassing key components such as generating, promoting, implementing creative ideas, materials and tools for instruction and evaluation. The purposes of these activities include promoting teaching*

effectiveness and fostering students' development. These various proposals suggest that teaching innovation is more than just incorporating new technologies into the classroom and promoting achievement; it also involves a new ideology that educates students to become independent and responsible learners and social agents. It is also worth noting that the studies agree that teachers' activities are embedded in interactive social and pedagogical environments. One's own teaching innovation should be intertwined with that of other members, although it could also be initiated at the individual level (Nguyen et al., 2021).

Innovation in teaching is associated with endogenous variables such as attitudes (Chang & Chen, 2015), intelligence and knowledge (Mishra & Koehler, 2006), and motivation (Lourmpas & Dakopoulou, 2014). It is also enhanced or affected by innovation atmosphere (Hou, 2018; Sagnak, 2012), work autonomy (Nguyen et al., 2021), organizational support (Cai & Tang, 2021; Mokhber et al., 2018), and most importantly, the principal's leadership style (Marks & Printy, 2003). Transformational (Bednall et al., 2018; Hallinger, 2003; Hou, 2018; Marks & Printy, 2003; Rashid & Halim, 2015) and transactional leadership (Hou, 2018) have been found to be positively associated with teachers' performance in developing and implementing creative ideas in schools. Decentralized leadership by school leaders encourages teachers to adopt network-based innovations in the workplace (Brown et al., 2020). Empowering leadership from school leaders (Gkorezis, 2016; Sagnak, 2012) encourages teachers to explore, which in turn facilitates their innovative work behaviors. Instructional leadership (Marks & Printy, 2003), visionary leadership (Chen & Yuan, 2021), and servant leadership (Chang et al., 2016) are also considered conducive to creative teaching behaviors.

2.2.3 The impact of temporal leadership on teaching innovation

Although there are no publications on the role of temporal leadership in innovation theory, existing studies in business and industry have shown that temporal leadership of team leaders is positively associated with innovative behavior (Liu et al., 2021; Xiao et al., 2020; Zhang et al., 2021) and creativity (Gevers & Demerouti, 2013), and facilitates business innovation, venturing, and strategic renewal activities (Chen & Nadkarni, 2017). From a team process perspective, temporal leadership positively impacts team transition, action, and interpersonal processes. Team transition describes the team's behaviors related to mission analysis, goal setting, and strategy formulation and planning. Team action assesses team members' engagement in accomplishing tasks, such as monitoring progress toward goals, backup behaviors, and coordination. Interpersonal processes assess the degree of trust and support team members share in resolving task and emotional conflicts (Maruping et al., 2015). When team leaders exhibit strong temporal leadership behaviors, they reduce time-related problems such as procrastination, decrease burnout and conflict (Chen & Liu, 2020), and ultimately increase diversity benefits in terms of time-based urgency and pace style (Mohammed & Alipour, 2014). Leaders who provide temporal coordination or support individuals in addressing time-related issues encourage teams to develop an integrated approach to time, thereby increasing task clarity (Fuerstenberg et al., 2021) and synchronizing organizational dynamics, which better positions the team for higher quality productivity (Santos et al., 2016).

With this in mind, it is plausible that temporal leadership can avoid team conflict and increase the temporal smoothness of the team, which would help teachers adjust their pace to external deadlines and increase efficiency. Temporal synergy in the team

could also minimize problems such as time-based fragmentation, time-based uncertainty, and time-based ambiguity, giving teachers more time-based resources and autonomy for innovative activities. Based on the JD-R model and previous findings, we propose that principals' temporal leadership, a type of organizational control, may act as a positive labor resource for teaching innovation. We, therefore, develop the first hypothesis of the current study:

H1*: Temporal leadership is positively predictive of teaching innovation.*

2.3 The mediation of job autonomy

2.3.1 Job autonomy

Previous studies on innovation in business and the economy show that the influence of temporal leadership is mediated by factors such as temporal conflicts (Chen & Liu, 2020), pro-social rule violation for efficiency (PSRB_E), and vitality (Zhang et al., 2021). Similarly, the effects on teaching innovation are also mediated by external factors. In this study, we choose to focus on workplace autonomy, one of the key workplace resources in influential theories of organization and work environment (Deci & Ryan, 2000; Karasek, 1979); however, its role in temporal leadership has been sparsely studied. Autonomy is one of the central motivating factors for organizational behavior and leadership in the work characteristics model (Fried & Ferris, 1987; Hackman & Oldham, 1976) and self-determination theory (Deci & Ryan, 2000; Ryan & Deci, 2000). It is expected that the inclusion of this factor will help uncover the mediating motivational mechanism underlying the effects of temporal leadership.

Autonomy in the workplace is also referred to as job control in other influential theories, such as demand control theory (Karasek, 1979). The concept of "autonomy" can be interpreted at the individual, collective, or state level (Nguyen et al., 2021). At

the individual level, which is the focus of this study, workplace autonomy reflects the amount of freedom a worker has in deciding what to do at work and how to do it (Deci & Ryan, 2000; Karasek, 1979; Kirmeyer & Shirom, 1986). The classic model of (Breaugh, 1985) defines three of its dimensions: Autonomy in terms of methods, timing, and criteria. Other researchers propose a four-dimensional model: Autonomy of work method, of work scheduling, of work time, and of work location (De Spiegelaere et al., 2016).

2.3.2 Job autonomy as an antecedent of innovative behavior

Autonomy in the workplace is a critical factor in employee engagement and innovative behavior (Chang et al., 2012; Kirmeyer & Shirom, 1986). It is a precondition for innovation (Hammond et al., 2011), and can have a direct effect on innovative work behaviors; however, it can also have an indirect effect by mediating job engagement, whereby job autonomy increases job engagement, which in turn promotes innovative work behaviors, such as idea generation and implementation (De Spiegelaere et al., 2014). Autonomy is thought to influence innovation not only through the degree of autonomy granted by organizations, but also through the autonomy individuals gain for themselves (Criscuolo et al., 2014). As a multidimensional construct, all of its dimensions are positively related to innovative behavior, with the role of autonomy in work methods and work locations being particularly significant (De Spiegelaere et al., 2016). Giving employees the freedom to determine how tasks should be performed can increase their willingness to develop and implement new ideas and technologies (Hammond et al., 2011).

In terms of teaching, autonomy also has a number of positive functions. It is a catalyst for teachers' professional competence, a source of effectiveness, and a source

of creativity, experimentation, and diversity (Cribb & Gewirtz, 2007). Teachers' weekly work autonomy, along with other work resources, positively correlates with weekly engagement, which in turn increases their work performance (Bakker & Bal, 2010). Job autonomy also influences teachers' collective innovativeness by mediating their participation in professional learning activities (Nguyen et al., 2021). In contrast, a lack of autonomy discourages teachers from engaging in innovative instructional activities, such as implementing new pedagogical approaches to teaching English (Benson, 2010).

2.2.3 The myth between temporal leadership and job autonomy

Autonomy in the workplace can be explained by management practices and leadership behaviors (Ferris, 1983; Kirmeyer & Shirom, 1986). It is promoted by leadership styles such as integrative (Shakil et al., 2021), ethical (Liu et al., 2021), and empowering leaderships (Zhang & Bartol, 2010). It is especially emphasized by laissez-faire leadership (Eagly et al., 2003). Ethical leaders provide employees with greater autonomy at work by providing social cues embedded in values, work requirements, and expectations (X. Liu et al., 2021). Transformational leadership also promotes employee autonomy at work, such that some studies (Goezuekara & Şimşek, 2015) found that the latter fully mediates its effect on work engagement. In contrast, workplace autonomy is impaired in leadership styles such as autocratic leadership (Eagly et al., 2003) or lean production (Rodríguez et al., 2016), which control all decisions and require little input from employees. In educational systems, teachers' workplace autonomy can be shaped by educational reforms (Taylor, 2007) and constrained by policies such as "Schemes of Work" set by local and national boards, the state, and school-based oversight and monitoring mechanisms (Benson, 2010).

While the pathway from workplace autonomy to teaching innovation is well

documented, the impact of temporal leadership on staff workplace autonomy remains unresolved. Since temporal leadership concerns managerial behavior in scheduling, allocating, and synchronizing subordinates' time resources, and while job autonomy requires freedom from external control, temporal leadership appears to affect job autonomy. However, the term "autonomy" in organizational management does not mean that one's work behavior can be completely isolated or independent of other team members. Unsynchronized time structures can lead to fragmented work and higher burnout. Indeed, workplace autonomy and team time management are not incompatible in modern knowledge work. One can enjoy strong autonomy while being bound to the macro team framework (Vaeaenaenen et al., 2020). Time is an important dimension of work autonomy. Effective time management by managers can avoid planning uncertainties and increase task clarity. Team members can then enjoy a high level of time commitment and gain more free time for innovative activities (Breaugh, 1985; De Spiegelaere et al., 2016; Ruubel, 2020; Vaeaenaenen et al., 2020). Therefore, we posit the following hypothesis:

H2*: Team temporal leadership promotes teachers' job autonomy.*

H3*: Job autonomy mediates the impact of temporal leadership on teaching innovation.*

2.4 The moderation of work stress

Because stress is ubiquitous in the workplace, it is a classic and fundamental work demand in models of organizational behavior. In the theory of job demands and resources (Karasek, 1979), job demand is measured by job performance pressures, psychological stressors associated with workload, unexpected tasks, work-related personal conflicts, unemployment or job anxiety, career development, and physical

stressors. Stress reflects "individuals' perceived demands versus resources" (Phillips-Wren & Adya, 2020). The American Institute of Stress defines stress as "a state or feeling experienced when a person perceives that demands exceed the personal and social resources that the individual can mobilize" and considers it an intervening variable with antecedent causes and behavioral consequences (Stress, 2020).

According to the theory of work demands and resources (Bakker & Demerouti, 2017; Demerouti et al., 2001), work demands and work resources interact to influence health, well-being, and work engagement. The hypothesis is that work resources are most useful for maintaining work engagement under conditions of high work demands (e.g., workload and unfavorable physical environment) (Bakker & Demerouti, 2014). Accordingly, the innovation-enhancing effect of temporal leadership is stronger when time pressure is high (Liu et al., 2021) or when the employee has a higher synchrony preference (Xiao et al., 2020). The effect of autonomy on innovation also depends on boundary conditions. It is modulated by working conditions, employees' prior experience or skills, and self-control (Chang et al., 2012). However, little is known about how the effects of temporal leadership and work autonomy on teaching innovation might be moderated by boundary conditions.

The literature has documented complex functions of work stress on organizational performance and innovation. Behavioral and psychological models have identified two typical copying behaviors under stress: People revert to rigid and routine responses from previous experiences (Shah et al., 2012) or make risky and erroneous decisions (Phillips-Wren & Adya, 2020). Stress is perceived by some researchers as an innovation killer. Under stress or when resources are scarce, people shift their attention to the status quo, reinforcing the urgency and importance of what is at hand at the expense of the future. Stress requires additional resources to maintain replenishing resources. It also

shifts individuals' attention to basic needs at the bottom of the hierarchy (e.g., safety, food, or drink) rather than needs at the top (e.g., self-actualization and transcendence) (Shah et al., 2012). However, occupational stress can also have a positive effect on innovative work behavior (Daniel et al., 2020). Individuals under stress may be compelled to seek solutions and overcome challenges in an active problem-solving mode to self-develop and transform (Anderson et al., 2004). Additionally, stress could lead to higher levels of motivation, learning, and innovative behavior (Karasek, 1989; Anderson et al., 2004).

Based on the above models and findings, we hypothesize the following:

H4: *Work stress modulates the effect of temporal leadership on teaching innovation: first, through the direct pathway of influence of temporal leadership on teaching innovation, and second, through the second half of the indirect mediation pathway between temporal leadership and teaching innovation via workplace autonomy.*

3. Methods

3.1 Participants

A total of 2021 valid samples were recruited from teachers in elementary or middle schools in 19 provinces in mainland China using a random cluster sampling method; including 436 men and 1,585 women with a mean age of 36.67 years (*SD* = 8.92). The response rate was 80.8%. In distributing the questionnaires, we treated the school unit as one group. First, the principal asked teachers to gather at a predetermined location. A trained research assistant then formulated the detailed guidelines, explained the implications of the survey, and ensured that anonymity was maintained. Teachers were

asked to respond to all questions in the questionnaire according to their natural feelings. Important socio-demographic variables such as gender, age, and education level, were also asked. The study was approved by the Ethic Committee of Xinyang Normal University (No. XYEC-2021-009), all the teachers gave written consents for participation.

3.2 Measurement and construct validation

3.2.1 Temporal leadership

Teachers were asked to rate their supervisor's temporal leadership, such as commitment to scheduling, synchronizing, and allocating temporal resources, using the scale developed by Myer and Mohammed (Myer, 2010; Myer & Mohammed, 2012). The scale contained 10 items, with five items for each of the two dimensions: task-oriented temporal leadership and relationship-oriented temporal leadership. A sample item for the task-oriented dimension was "To what extent does your team leader push members to complete subtasks on time?" and a sample item for the relationship-oriented dimension was "To what extent does your team leader praise (commend) team members for completing work on time?" Each item was scored on a 5-point scale, ranging from 1 (strongly disagree) to 5 (strongly agree), with a higher score indicating a higher level of team temporal leadership. The Cronbach's α for this scale was 0.929 for the current sample.

3.2.2 Teaching innovation

We measured teaching innovation using Chen's questionnaire (Chen, 2009) for primary and secondary teachers as a reference. There were five dimensions, including innovation of ideas and thinking, of teaching content, of teaching methods, of teaching

materials, and of multiple assessment. Hou (2017) later adapted and revised the 25-item questionnaire by adding the specific situation of teachers. As the innovation in teaching community is fast evolving and some strategies listed in Hou (2017) 's questionnaire were not innovative any more, we consulted three experts in the education and management and deleted three items that did not fit for our targeting participants based on their consensus. We then tested the reliability and validity of the questionnaire for use in the context of rural education. The final version of the questionnaire comprised 22 items scored on a 5-point scale, with higher scores indicating higher teaching innovation. Confirmatory factorial analysis of all 2017 participants repeated the five similar dimensions of teaching innovation defined by Hou's (2018), including "thinking innovation, content innovation, method innovation, resource innovation, and assessment innovation". It also revealed a satisfactory fit to the five-factor model, chi-square = 2340.663, df = 199, $p < 0.001$, NFI = 0.926, RFI = 0.906, IFI = 0.932, TLI = 0.913, CFI = 0.932, RMSEA = 0.073. For the current sample, the Cronbach's α was 0.956.

3.2.3 Job autonomy

The 7-item scale developed by Kirmeyer and Shirom (1986) was used to assess perceived work autonomy. There were seven items rated on a 5-point scale, with higher scores indicating higher levels of autonomy at work. An example item was "I can decide how to perform my job based on my work style." For the current sample, the Cronbach's α was 0.868.

3.2.4 Work stress

The extent to which employees felt stressed was measured using the scale

developed by Cavanaugh et al. (2000), which contained 11 items on two dimensions: challenge-related stress and obstacle-related stress. One sample item was, “I number of projects or tasks I have.”" Cronbach’s α was 0.929 for the present sample.

3.3 Data processing

Statistical analyzes were performed using IBM SPSS 20.0 software in the following steps: (1) descriptive statistics and correlation analysis for each variable; (2) common method bias analysis;(3) regression analyses to assess the mediation of job autonomy on the effect of temporal leadership on teaching innovation; and (4) a conditional process analysis was then performed to examine the modulation of job stress on the mediated effect of temporal leadership.

4. Results

4.1 Common method bias

To minimize the possibility of questionnaire bias (common method bias, CMB) (Podsakoff et al., 2003), we anonymized all respondents and used one-factor method to test for CMB. We included all items of the above-mentioned 4 questionnaires and performed a principal component analysis. The results revealed two factors with eigenvalues greater than one. The first factor extracted explained 27.57% of the total variation before rotation and 21.6% after rotation, which was far below the critical value of 40% and suggested no obvious CMB.

4.2 Mean values, standard deviations, and correlation matrixes for each variable

Table 1 presents the means, standard deviations, and correlation matrices for each

variable. We found that gender, age, and educational background were significantly correlated with some variables. Therefore, we controlled for each of these variables in a subsequent regression analysis, finding significant correlations with temporal leadership in all cases. Specifically, temporal leadership was significantly and positively correlated with teaching innovation and work autonomy, but negatively correlated with work stress. Additionally, there was a critical correlation between teaching innovation and work stress. Finally, there was a significant and negative correlation between work autonomy and work stress.

Table 1. Mean values, standard deviations, and correlation matrixes among variables

	M±*SD*	1	2	3	4	5
1. Gender	0.79±0.411	—				
2. Age	36.77±8.714	-0.331***	—			
3. Temporal leadership	3.404±0.813	0.072**	-0.097***	—		
4. Job autonomy	3.359±0.719	0.017	-0.025	0.403***	—	
5. Work stress	3.152±0.796	-0.122***	0.035	-0.170***	-0.227***	—
6. Teaching innovation	4.171±0.466	0.098***	0.031	0.303***	0.244***	-0.069**

Note: * p < 0. 05, **p < 0. 01, ***p < 0. 001. Gender was the dummy variable. Female = 0, Male = 1. The mean value represents the proportion of females.

4.3 Test of the conditional process model with work stress as a moderator

All variables were standardized to avoid multicollinearity. We then tested Hypothesis 1 through a linear multiple regression analysis with teaching innovation as the dependent variable and team temporal leadership over time as the independent variable, while the control covariates included age and gender. The results showed a significant positive prediction of temporal leadership on teaching innovation (β = 0.305,

t=*14.111*, $p < 0.001$). We then constructed a mediation model (Model 4) using ordinary least squares path analysis in PROCESS 3.0 (Hayes, 2018; Hayes & Rockwood, 2020) to test Hypotheses 2 and 3. The significance of the indirect effect was estimated using 5000-percentile bootstrap samples, and the mediation effect size was indexed by the partially standardized indirect effect (Fig 1). As predicted, temporal leadership was positively predictive of job autonomy ($\beta_a = 0.404$, $t = 19.683$, $p < 0.001$, [95% bootstrapped CI: 0.321 to 0.392]) and job autonomy was positively associated with teaching innovation ($\beta_b = 0.148$, $t = 6.482$, $p < 0.001$, [CI: 0.677 to 0.126]). This resulted in a partial but significant mediation effect of job autonomy ($ab = 0.060$, $p < 0.05$, [CI: 0.040 to 0.080]), leaving the direct effect of temporal leadership on teaching innovation $c' = 0.245$ ($t = 10.726$, $p < 0.001$).

Fig 1. job autonomy mediates the relationship between temporal leadership and reaching innovation

--

We then tested the moderated mediation effect of work stress (Fig 2). Model 15 was chosen to test the hypothesis that work stress influences the direct effect of temporal leadership on teaching innovation as well as the second part of the mediation pathway. Regression analysis revealed the positive relationship between job autonomy and teaching innovation ($\beta = 0.399$, $t = 5.055$, $p < 0.001$, [CI: 0.244 to 0.553]). The main effect of work stress on teaching innovation was not significant ($\beta = 0.039$, $t = 1.441$, $p > 0.092$ [CI: -0.014 to 0.092]). However, the magnitude of the interaction between work stress and work autonomy was significantly negatively associated with teaching innovation ($\beta = -0.076$, $t = -3.224$, $p = 0.001$, [CI: -0.122 to -0.030]). These results support Hypothesis 4, that work stress modulates the second limb of the

mediation effect of occupational autonomy. Meanwhile, we observed a significant modulating effect of work stress on the direct effect of temporal leadership on teaching innovation, as their interaction was significantly and negatively associated with teaching innovation (β = -0.079, t = -3.094, p = 0.002, [CI: -0.129 to -0.029]).

Fig 2. The moderation of work stress

We then constructed slope diagrams to visualize the modulation effect of work stress. Previous studies have reported complex profiles, either an inverted U-shape or a linear shape, of the effect of stress on work performance. Taking this into consideration, we categorized work stress into three levels: high, medium, and low level groups, based on standard deviations -1, 0, and 1 from the mean. The influence of temporal leadership on teaching innovation varied among the different stress levels, as did the influence of job autonomy on teaching innovation. There are significant conditional effects of temporal leadership at all three stress levels (Fig 3): lower stress (*effect* = 0.308, t=10.199, p < 0.001, [CI: 0.249, 0.367]), medium stress (*effect* = 0.245, t=10.548, p < 0.001, [CI: 0.200, 0.290]), higher stress (*effect* = 0.182, t=5.886, p < 0.001, [CI: 0.121, 0.243]). However, the significant interaction implies that the conditional effect of temporal leadership is largest in the lower stress situation, and smallest in the higher stress situation. This indicates that temporal leadership facilitates teaching innovation at all three stress levels; however, the facilitation effect decreases as work stress increases.

Fig 3. Simple slope analysis on the moderation of work stress

----

There are significant conditional effects of job autonomy in all three levels of

stress: lower stress (*effect* = 0.089, [CI: 0.059, 0.120]), middle stress (*effect* = 0.065, [CI: 0.044, 0.089]), higher stress (*effect* = 0.040, [CI: 0.013, 0.068]), leading to a significant moderated mediation of these two factors, *effect* = -0.031, [CI: -0.057, -0.008]). The conditional effect of temporal leadership is largest in the lower stress situation and smallest in the higher stress situation. This implies that workplace autonomy promotes teaching innovation at all three stress levels; however, the promoting effect decreases as work stress increases.

5. Discussion

Our findings reveal a clear message: **we may not be able to schedule teaching innovation, but we can schedule for it.** Teaching innovation is a necessity to meet the needs of the modern world with its ever-changing technologies and increasing time pressures and stress. Although time has emerged as an important dimension in renovated leadership theories (Fischer et al., 2017) and is perceived as a critical factor that continuously impacts innovation (Amabile et al., 2002), few empirical studies exist on the relationship between temporal leadership and teaching innovation. To the best of our knowledge, this study is the first to link the two important constructs through a questionnaire survey of a representative sample of primary and secondary teachers. The results show a significant positive direct effect of temporal leadership on teachers' innovative work behavior. Temporal leadership facilitates teaching innovation via the mediation of job autonomy; it also facilitates job autonomy, which, in turn, enables teaching innovation. In addition, both the direct and indirect effects are susceptible to modulating work stress. These findings are an important contribution to educational management and temporal leadership literature and have important theoretical and practical implications.

5.1 Theoretical Implications

The first important finding is that the positive effect shows a significant positive impact on teaching innovation. This is consistent with previous findings that innovative instruction would benefit from factors such as management support, administrative support, and district coordinator support and guidance (Thurlings et al., 2015). It is also consistent with findings that team temporal leadership positively affects innovation and work performance (Chen & Nadkarni, 2017; Gevers & Demerouti, 2013; Halbesleben et al., 2003; Mudannayake et al., 2016) in business and economics. Our work represents an important extension of research in temporal leadership to the field of innovation in education. It also suggests cross-domain consistency regarding the impact of temporal leadership on innovation behavior.

While replicating previous effects of job autonomy on teaching innovation (Benson, 2010; Nguyen et al., 2021), our study shows, for the first time, that temporal leadership favors job autonomy. The importance of autonomy for creativity and innovation is well known. Freedom from external pressures stimulates interest in the task itself, increases intrinsic motivation, and thus promotes innovative behavior (Deci & Ryan, 2000). Therefore, it is important to explore factors and approaches that can promote autonomy in the workplace. Previous research on promoting autonomy has focused on strategies, such as empowering employees to make independent decisions (Costa et al., 2016), encouraging exploration and innovation (Tu & Lu, 2013), and effective leadership style (Goezuekara & Şimşek, 2015; X. Liu et al., 2021; Shakil et al., 2021). Although time is critical to the management of an organization and time-based autonomy is a core dimension of job autonomy, little is known about the relationship between team leaders' temporal leadership and employees' job autonomy,

because the concept of temporal leadership is relatively new. This finding also contributes to the field of temporal leadership by enriching its functions.

Autonomy means the freedom to make decisions and implement ideas without being constrained by external pressures (Deci & Ryan, 2000; Karasek, 1979; Kirmeyer & Shirom, 1986). Because temporal leadership of teams involves control behaviors on the part of managers, such as scheduling and time allocation, as well as dealing with individual differences in task pace, one might speculate that temporal leadership, which involves demands and controls from organizational management and makes employees less independent, may impair autonomy and innovation. In contrast to this hypothesis, however, our results show a facilitative effect of temporal leadership, suggesting that control and autonomy are not mutually exclusive, but can coexist. Recently, the interesting topic of bounded autonomy in knowledge work has emerged, and studies are called for to explore the methods by which it can be established. Researchers argue that since the work process is embedded in multiple social and organizational relationships, "a high degree of individual decision-making autonomy and a high degree of connectivity and interdependence must coexist" (Vaeaenaenen & Toivanen, 2018). Our study is a response to this call by suggesting that temporal leadership can be used to realize bounded autonomy in organizations.

Temporal leadership is not synonymous with rigid time management that turns a blind eye to individual differences among team members. According to the theoretical constructs of Mohammed and Alipour (2014), and Myer and Mohammed (2012), effective temporal leadership involves planning and allocating time resources with full consideration of individual differences in time-based orientation, pace, rhythm, and task difficulty, as well as appreciating and encouraging team members as they grapple with time-related work problems. Here, we propose two possible ways in which effective

temporal leadership promotes work autonomy: 1) temporal leadership provides employees with freer time resources by minimizing time fragments and increasing task clarity; and 2) temporal leadership resolves team conflicts, thereby ensuring smooth teamwork and bound autonomy that is fair to both members and the organization. Future studies can explore this proposition to uncover the processes underlying the effects of temporal leadership on work autonomy.

The modulation of work stress describes a boundary condition for the effects of temporal leadership and job autonomy on teaching innovation. It also supports the theory of job demands and resources (Bakker & Demerouti, 2014; Demerouti et al., 2001) and the theory of job demands and control (Karasek, 1979) by including temporal leadership as an element of job demands. Our study shows that work stress modulates the direct impact of temporal leadership on teaching innovation. Work stress also modulates the effect of job autonomy on teaching innovation; in other words, the second side of the mediating path from temporal leadership to teaching innovation via job autonomy. The results showed that the benefits of higher temporal leadership on teaching innovation were smaller at higher levels of stress, but much larger at lower levels of stress. This suggests that the benefits of temporal leadership for teaching innovation become smaller as work stress increases. Previous studies found that a higher effect of temporal leadership was mediated by the amount of temporal conflict in the group. Benefits were higher when group temporal conflict was low, but decreased as time-based conflict increased (Najam et al., 2018). When time-based diversity is higher, higher temporal leadership leads to the highest time-based conflict, while higher temporal leadership leads to the lowest time-based conflict when time-based diversity is lower (Najam et al., 2018). When team temporal leadership is strong, the indirect effect of perceived time pressure on team performance is usually positive, whereas

when team temporal leadership is weak, the indirect effect is positive when perceived time pressure is low, and negative when perceived time pressure is moderate to high (Maruping et al., 2015).

5.2 Practical Implications

Our findings provide three practical suggestions for educational management and, more broadly, leadership. First, our study clearly shows that temporal leadership can be useful for school leaders. It can be used to promote job autonomy and support innovative work behaviors among their staff, such as teaching innovation. Leaders can use levers, such as setting deadlines for tasks and encouraging their staff to stick to the schedule, to avoid time pressure to innovate (Amabile et al., 2002). It is important that managers avoid rigid task control when performing these activities; rather they should adapt their management practices to individual differences in time use (Gevers & Demerouti, 2013). In other words, they should pay attention to individual differences in team members' time orientation, pace, skills, and time urgency. Team members' appreciation of their time-related activities is also important. These findings are also significant in selecting team members when assembling the group. Selecting faculty with shared temporal cognition would be helpful because there is evidence that shared temporal cognition can, to some extent, substitute for the effect of temporal leadership on team work performance (Abrantes et al., 2018; Santos et al., 2016). Second, when using team temporal leadership as a catalyst for teaching innovation, leaders should be aware of the modulation of stress. Our results show that the effect size of temporal leadership is largest at lower levels of work stress, but smallest at higher levels of work stress. This means that leaders can use temporal leadership to promote teaching innovation at any stress level, but they should adjust their expectations accordingly.

Third, leaders are advised to pay attention to motivational stimuli when promoting innovative behavior through team time management. Our study shows that workplace autonomy is a positive predictor of teaching innovation and that it also partially mediates the effect of temporal leadership on teaching innovation. This implies that in order to promote teaching innovation, school leaders can mobilize factors that promote workplace autonomy and thus could magnify the outcome of their leadership behavior. It is expected that this would also be true for innovative activities in other areas.

5.3 Limitations and Further Directions

Despite these innovations and strengths, the results of our study should be interpreted with caution. First, our study is based on observations of primary and secondary school teachers in mainland China. Chinese culture emphasizes social harmony, conformity, and collective thinking, which is quite different from Western cultures, where individual contribution is more valued. There are also significant differences between countries in the systems of educational administration and pedagogy. For example, teachers in different countries have a different conception of teacher autonomy and a different understanding of pedagogical tasks (Silva, 2021). It is also worth noting that the professional characteristics of teachers differ from other organizations, such as companies and firms, where innovative behaviors are more emphasized. The activities in the school system are largely routine, as the main task of teachers is to teach, as prescribed by the school board, and teaching innovation is only a small part of their task. Thus, the question remains as to whether the same relationship between temporal leadership and job autonomy, temporal leadership and innovative behavior can be observed in other cultures, educational systems, and social sectors. Future studies are needed to clarify these issues. Additionally, previous studies have

found differential effects of leadership behavior on subjective job performance that are not objectively measured (e.g., third-party evaluation or quantity or quality of products). In our study, teaching innovation behavior was measured by teachers' self-assessment. Finally, innovation requires autonomy. Some studies have shown that innovation can be hindered when behavioral norms are strictly enforced or when lesson plans are far too detailed (Criscuolo et al., 2014). Thus, the question arises as to what constitutes effective temporal leadership and what the difference is between the quality and the intensity of temporal leadership. Future studies are needed to make these distinctions.

References

Abrantes, A. C. M., Passos, A. M., Cunha, M. P. e., & Santos, C. M. (2018). Bringing team improvisation to team adaptation: The combined role of shared temporal cognitions and team learning behaviors fostering team performance. *Journal of Business Research, 84,* 59-71. doi:https://doi.org/10.1016/j.jbusres.2017.11.005

Alipour, K. K., Mohammed, S., & Martinez, P. N. (2017). Incorporating temporality into implicit leadership and followership theories: Exploring inconsistencies between time-based expectations and actual behaviors. *The Leadership Quarterly, 28*(2), 300-316. doi:https://doi.org/10.1016/j.leaqua.2016.11.006

Amabile, T. M., Hadley, C. N., & Kramer, S. J. (2002). Creativity Under the Gun. *Harvard Business Review, 80*(8), 52-61, 147.

Ancona, D. G., Goodman, P. S., Lawrence, B. S., & Tushman, M. L. (2001). Time: A New Research Lens. *Academy of Management Review, 26*(4), 645-663. doi:10.5465/amr.2001.5393903

Bakker, A. B., & Bal, M. P. (2010). Weekly work engagement and performance: A study among starting teachers. *Journal of Occupational and Organizational Psychology, 83*(1), 189-206. doi:10.1348/096317909X402596

Bakker, A. B., & Demerouti, E. (2007). The job demands-resources model : state of the art. *Journal of Managerial Psychology, 22*(3), 309-328. doi:10.1108/02683940710733115

Bakker, A. B., & Demerouti, E. (2014). Job Demands–Resources Theory. In P. Y. Chen & C. L. Cooper (Eds.), *Work andWellbeing: Wellbeing: A Complete Reference Guide, Volume III* (pp. 1-28): John Wiley & Sons, Inc.

Bakker, A. B., & Demerouti, E. (2017). Job demands-resources theory: taking stock and looking forward. *Journal of Occupational and Organizational Psychology, 22*(3), 273-285. doi:10.1037/OCP0000056

Bakker, A. B., Hakanen, J. J., Demerouti, E., & Xanthopoulou, D. (2007). Job Resources Boost Work

Engagement, Particularly When Job Demands Are High. *journal of educational psychology, 99*(2), 274-284. doi:10.1037/0022-0663.99.2.274

Bednall, T. C., E. Rafferty, A., Shipton, H., Sanders, K., & J. Jackson, C. (2018). Innovative Behaviour: How Much Transformational Leadership Do You Need? *British Journal of Management, 29*(4), 796-816. doi:https://doi.org/10.1111/1467-8551.12275

Benson, P. (2010). Teacher education and teacher autonomy: Creating spaces for experimentation in secondary school English language teaching. *Language Teaching Research, 14*(3), 259-275. doi:10.1177/1362168810365236

Blândul, V. C. (2015). Inovation in Education – Fundamental Request of Knowledge Society. *Procedia - Social and Behavioral Sciences, 180*, 484-488. doi:10.1016/J.SBSPRO.2015.02.148

Breaugh, J. A. (1985). The measurement of work autonomy. *human relations, 38*(6), 551-570. doi:10.1177/001872678503800604

Brown, C., MacGregor, S., & Flood, J. (2020). Can models of distributed leadership be used to mobilise networked generated innovation in schools? A case study from England. *Teaching and Teacher Education, 94*, 103101. doi:https://doi.org/10.1016/j.tate.2020.103101

Cai, Y., & Tang, R. (2021). School support for teacher innovation: Mediating effects of teacher self-efficacy and moderating effects of trust. *Thinking Skills and Creativity, 41*, 100854. doi:https://doi.org/10.1016/j.tsc.2021.100854

Cao, C., Shang, L., & Meng, Q. (2020). Applying the Job Demands-Resources Model to exploring predictors of innovative teaching among university teachers. *Teaching and Teacher Education, 89*, 103009. doi:https://doi.org/10.1016/j.tate.2019.103009

Cavanaugh, M. A., Boswell, W. R., Roehling, M. V., & Boudreau, J. W. (2000). An empirical examination of self-reported work stress among U.S. managers. *Journal of Applied Psychology, 85*(1), 65-74. doi:10.1037/0021-9010.85.1.65

Chang, C.-P., & Chen, I. J. (2015). Correlation between Pre-Service Teachers' Information Technology Integration Attitude and Creative Teaching Behavior. *creative education, 6*(16), 1802-1814. doi:10.4236/CE.2015.616184

Chang, C.-P., Tsai, H.-C., Zhang, D.-D., & Chen, I.-J. (2016). The Correlation between Elementary School Principals¡¯ Servant Leadership and Teachers¡¯ Creative Teaching Behavior. *creative education, Vol.07No.09*, 8. doi:10.4236/ce.2016.79132

Chang, J., Huang, D., & Choi, J. (2012). Is Task Autonomy Beneficial for Creativity? Prior Task Experience and Self-Control as Boundary Conditions. *Social Behavior and Personality: an international journal, 40*. doi:10.2224/sbp.2012.40.5.705

Chen, H.-H., & Yuan, Y.-H. (2021). The Study of the Relationships of Teacher's Creative Teaching, Imagination, and Principal's Visionary Leadership. *SAGE Open, 11*(3), 21582440211029932. doi:10.1177/21582440211029932

Chen, J., & Liu, L. (2020). Reconciling temporal conflicts in innovation ambidexterity: the role of TMT temporal leadership. *Journal of Knowledge Management, 24*(8), 1899-1920. doi:10.1108/JKM-10-2019-0555

Chen, J., & Nadkarni, S. (2017). It's about Time! CEOs' Temporal Dispositions, Temporal Leadership, and Corporate Entrepreneurship. *Administrative Science Quarterly, 62*(1), 31-66. doi:10.1177/0001839216663504

Chen, S.-T. (2009). *A Study of the Relations between Innovative Teaching Capability and Teaching*

Effectiveness of Teachers in Elementary and Junior High Schools in Penghu County. (Master). National Taipei University of Education, Taipei.

Chen, Y.-S., & Hu, M.-C. (2008). The Impact of Task Motivation and Organizational Innovative Climate On Adult Education Teachers' Creative Teaching Performance: An Analysis of Hierachical Linear Modeling. *Bulletin of Educational Psychology, 40*(2), 179-198.

Costa, S., Cuzzocrea, F., Gugliandolo, M. C., & Larcan, R. (2016). Associations Between Parental Psychological Control and Autonomy Support, and Psychological Outcomes in Adolescents: The Mediating Role of Need Satisfaction and Need Frustration. *Child Indicators Research, 9*(4), 1059-1076. doi:10.1007/s12187-015-9353-z

Cribb, A., & Gewirtz, S. (2007). Unpacking Autonomy and Control in Education: Some Conceptual and Normative Groundwork for a Comparative Analysis. *European Educational Research Journal, 6*(3), 203-213. doi:10.2304/eerj.2007.6.3.203

Criscuolo, P., Salter, A., & Wal, A. L. J. T. (2014). Going Underground: Bootlegging and Individual Innovative Performance. *Organization Science*, *25*(5), 1287-1305. doi:10.1287/orsc.2013.0856

Daniel, J. L., Chatelain-Jardon, R., & Zhuofan, Z. (2020). Does Stress Lead to Creativity?: The Relationship Between Occupational Stress and Individual Innovative Behavior. *Studies in Business and Economics, 15,* 21-30. doi:10.2478/sbe-2020-0003

De Spiegelaere, S., Van Gyes, G., De Witte, H., Niesen, W., & Van Hootegem, G. (2014). On the Relation of Job Insecurity, Job Autonomy, Innovative Work Behaviour and the Mediating Effect of Work Engagement. *Creativity and Innovation Management, 23*(3), 318-330. doi:https://doi.org/10.1111/caim.12079

De Spiegelaere, S., Van Gyes, G., & Van Hootegem, G. (2016). Not All Autonomy is the Same. Different Dimensions of Job Autonomy and Their Relation to Work Engagement & Innovative Work Behavior. *Human Factors and Ergonomics in Manufacturing & Service Industries, 26*(4), 515-527. doi:https://doi.org/10.1002/hfm.20666

Deci, E. L., & Ryan, R. M. (2000). The "What" and "Why" of Goal Pursuits: Human Needs and the Self-Determination of Behavior. *psychological inquiry, 11*(4), 227-268. doi:10.1207/S15327965PLI1104_01

Demerouti, E., Bakker, A. B., Nachreiner, F., & Schaufeli, W. B. (2001). The Job Demands-Resources Model of Burnout. *Journal of Applied Psychology, 86*(3), 499-512.

Eagly, A. H., Johannesen-Schmidt, M. C., & Engen, M. L. v. (2003). Transformational, Transactional, and Laissez-Faire Leadership Styles: A Meta-Analysis Comparing Women and Men. *Psychological Bulletin, 129*(4), 569-591. doi:10.1037/0033-2909.129.4.569

Ferris, G. R. (1983). The Influence of Leadership on Perceptions of Job Autonomy. *The Journal of Psychology, 114*(2), 253-258. doi:10.1080/00223980.1983.9915422

Fischer, T., Dietz, J., & Antonakis, J. (2017). Leadership process models: A review and synthesis. *journal of management, 43*(6), 1726-1753. doi:10.1177/0149206316682830

Foti, R. J., Hansbrough, T. K., Epitropaki, O., & Coyle, P. T. (2017). Dynamic viewpoints on implicit leadership and followership theories: Approaches, findings, and future directions. *The Leadership Quarterly*, *28*(2), 261-267. doi:10.1016/j.leaqua.2017.02.004

Fried, Y., & Ferris, G. R. (1987). The Validity of The Job Characteristics Model: A Review and Meta-Analysis. *Personnel Psychology, 40*(2), 287-322. doi:10.1111/J.1744-6570.1987.TB00605.X

Fürstenberg, N., Alfes, K., & Kearney, E. (2021). How and When Paradoxical Leadership Benefits Work

Engagement: The Role of Goal Clarity and Work Autonomy. *Journal of Occupational and Organizational Psychology, 94*(3), 672-705. doi:https://doi.org/10.1111/joop.12344

Gevers, J. M. P., & Demerouti, E. (2013). How Supervisors' Reminders Relate to Subordinates' Absorption and Creativity. *Journal of Managerial Psychology, 28*(6), 677-698. doi:10.1108/JPM-09-2011-0055

Gkorezis, P. (2016). Principal empowering leadership and teacher innovative behavior: a moderated mediation model. *International Journal of Educational Management, 30*(6), 1030-1044. doi:10.1108/IJEM-08-2015-0113

Goldsmith, R. E. (1986). Convergent Validity of Four Innovativeness Scales. *educational and psychological measurement, 46*(1), 81-87. doi:10.1177/0013164486461007

Gözükara, İ., & Şimşek, Ö. (2015). Linking Transformational Leadership to Work Engagement and the Mediator Effect of Job Autonomy: A Study in a Turkish Private Non-Profit University. *Procedia - Social and Behavioral Sciences, 195*, 963-971. doi:10.1016/j.sbspro.2015.06.274

Hackman, J. R., & Oldham, G. R. (1976). Motivation through the Design of Work: Test of a Theory. *organizational behavior and human performance, 16*(2), 250-279. doi:10.1016/0030-5073(76)90016-7

Halbesleben, J., Novicevic, M. M., Harvey, M. G., & Buckley, M. R. (2003). Awareness of temporal complexity in leadership of creativity and innovation: A competency-based model. *The Leadership Quarterly, 14*(4-5), 433-454. doi:10.1016/S1048-9843(03)00046-8

Hallinger, P. (2003). Leading Educational Change: reflections on the practice of instructional and transformational leadership. *cambridge journal of education, 33*(3), 329-352. doi:10.1080/0305764032000122005

Hammond, M., Neff, N., Farr, J., Schwall, A., & Zhao, X. (2011). Predictors of Individual-Level Innovation at Work: A Meta-Analysis. *Psychology of Aesthetics, Creativity, and the Arts, 5*, 90-105. doi:10.1037/a0018556

Hayes, A. F. (2018). *Introduction to mediation, moderation, and conditional process analysis: A regression-based approach (2nd ed.)*: New York, NY: Guilford Press.

Hayes, A. F., & Rockwood, N. J. (2020). Conditional Process Analysis: Concepts, Computation, and Advances in the Modeling of the Contingencies of Mechanisms. *American Behavioral Scientist, 64*(1), 19-54. doi:10.1177/0002764219859633

Hou, H. (2017). *The Effects of Transformational Leadership and Transactional Leadership on Teachers' Teaching Innovation.* (Master). Southwest University, Chongqing, China.

Hou, H. (2018). Can Principal Leadership Influence Teachers' Teaching Innovation? - On the Mediating Effect of Schools' Organizational Innovation. *Education Science (In Chinese), 34*(1), 26-32.

Hung, C.-L., & Li, F.-C. (2017). Teacher perceptions of professional role and innovative teaching at elementary schools in Taiwan. *educational research review, 12*(21), 1036-1045. doi:10.5897/ERR2017.3373

Karasek, R. A. (1979). Job Demands, Job Decision Latitude, and Mental Strain: Implications for Job Redesign. *Administrative Science Quarterly, 24*(2), 285-308. doi:10.2307/2392498

Keane, T., Boden, M., Chalmers, C., & Williams, M. (2020). Effective principal leadership influencing technology innovation in the classroom. *Education and Information Technologies, 25*(6), 5321-5338. doi:10.1007/s10639-020-10217-0

Kirmeyer, S. L., & Shirom, A. (1986). Perceived Job Autonomy in the Manufacturing Sector: Effects of

Unions, Gender, and Substantive Complexity. *Academy of Management Journal, 29*(4), 832-840. doi:10.5465/255949

Lattrich, K.-K., & Büttgen, M. (2020). Project Leaders' Control Resources and Role Overload as Predictors of Project Success: Developing The Job Demands–Resources Model. *business research, 13*(2), 767-788. doi:10.1007/S40685-020-00115-Z

Liu, X., Baranchenko, Y., An, F., Lin, Z., & Ma, J. (2021). The impact of ethical leadership on employee creative deviance: the mediating role of job autonomy. *Leadership & Organization Development Journal, 42*(2), 219-232. doi:10.1108/LODJ-01-2020-0026

Liu, Z., Liu, X., & Zhang, X. (2021). How to Solve the Time Dilemma? The Influence of Team Temporal Leadership on Team Innovation Performance. *Frontiers in Psychology, 12*(3025). doi:10.3389/fpsyg.2021.634133

Lourmpas, S., & Dakopoulou, A. (2014). Educational Leaders and Teachers' Motivation for Engagement in Innovative Programmes. The Case of Greece. *Procedia - Social and Behavioral Sciences, 116*, 3359-3364. doi:https://doi.org/10.1016/j.sbspro.2014.01.764

Marks, H., & Printy, S. (2003). Principal Leadership and School Performance: An Integration of Transformational and Instructional Leadership. *Educational Administration Quarterly, 39*, 370-397. doi:10.1177/0013161X03253412

Maruping, L. M., Venkatesh, V., Thatcher, S. M. B., & Patel, P. C. (2015). Folding Under Pressure or Rising to the Occasion? Perceived Time Pressure and the Moderating Role of Team Temporal Leadership. *Academy of Management Journal, 58*(5), 1313-1333. doi:10.5465/amj.2012.0468

Mishra, P., & Koehler, M. J. (2006). Technological Pedagogical Content Knowledge: A Framework for Teacher Knowledge. *teachers college record, 108*(6), 1017-1054. doi:10.1111/J.1467-9620.2006.00684.X

Mohammed, S., & Alipour, K. K. (2014). It's Time for Temporal Leadership: Individual, Dyadic, Team, and Organizational Effects. *Industrial and Organizational Psychology, 7*(2), 178-182. doi:https://doi.org/10.1111/iops.12128

Mohammed, S., & Nadkarni, S. (2011). Temporal Diversity and Team Performance: The Moderating Role of Team Temporal Leadership. *Academy of Management Journal, 54*(3), 489-508. doi:10.5465/amj.2011.61967991

Mokhber, M., Khairuzzaman, W., & Vakilbashi, A. (2018). Leadership and innovation: The moderator role of organization support for innovative behaviors. *Journal of Management & Organization, 24*(1), 108-128. doi:10.1017/jmo.2017.26

Mudannayake, B., Bhanugopan, R., & Lockhart, P. (2016). *The impact of team temporal leadership on employee engagement and team performance*. Paper presented at the 30th Australian and New Zealand Academy of Management Conference.

Myer, A. T. (2010). *Team Temporal Leadership: Construct Development and Validation.* (Master Degree). The Pennsylvania State University,

Myer, A. T., & Mohammed, S. (2012). *Team temporal leadership: Construct development and validation*. Paper presented at the 27th Annual Conference of the Society for Industrial and Organizational Psychology, San Diego, CA.

Najam, U., Inam, A., Awan, H. M., & Abbas, M. (2018). The Interactive Role of Temporal Team Leadership in the Telecom Sector of Pakistan: Utilizing Temporal Diversity for Sustainable Knowledge Sharing. *Sustainability, 10*(5), 1309. Retrieved from https://www.mdpi.com/2071-

1050/10/5/1309

Nguyen, D., Pietsch, M., & Gümüş, S. (2021). Collective teacher innovativeness in 48 countries: Effects of teacher autonomy, collaborative culture, and professional learning. *Teaching and Teacher Education, 106*, 103463. doi:https://doi.org/10.1016/j.tate.2021.103463

Phillips-Wren, G., & Adya, M. (2020). Decision Making Under Stress: The Role of Information Overload, Time Pressure, Complexity, And Uncertainty. *Journal of Decision Systems*, 1-13. doi:10.1080/12460125.2020.1768680

Podsakoff, P. M., MacKenzie, S. B., Lee, J. Y., & Podsakoff, N. P. (2003). Common method biases in behavioral research: a critical review of the literature and recommended remedies. *Journal of Applied Psychology, 88*(5), 879-903. doi:10.1037/0021-9010.88.5.879

Rashid, N. R. N. A., & Halim, N. A. (2015). Innovative Behavior in Educational Institutions : The Role of Transformational Leadership and Teamwork Attitude. *Journal of Management Sciences, 1*(1), 39-61.

Rinkevich, J. L. (2011). Creative Teaching: Why it Matters and Where to Begin. *The Clearing House, 84*(5), 219-223. doi:10.1080/00098655.2011.575416

Rodríguez, D., Buyens, D., Van Landeghem, H., & Lasio, V. (2016). Impact of Lean Production on Perceived Job Autonomy and Job Satisfaction: An Experimental Study. *Human Factors and Ergonomics in Manufacturing & Service Industries, 26*(2), 159-176. doi:https://doi.org/10.1002/hfm.20620

Ruubel, R. (2020). Time Dimensions of Job Autonomy in Estonian R&D Institutions. *Journal of the Knowledge Economy*. doi:10.1007/s13132-020-00633-5

Ryan, R. M., & Deci, E. L. (2000). Self-determination theory and the facilitation of intrinsic motivation, social development, and well-being. *American Psychologist, 55*(1), 68-78. doi:10.1037//0003-066X.55.1.68

Sagnak, M. (2012). The empowering leadership and teachers' innovative behavior: The mediating role of innovation climate. *African Journal of Business Management, 6*. doi:10.5897/AJBM11.2162

Santos, C. M., Passos, A. M., Uitdewilligen, S., & Nübold, A. (2016). Shared temporal cognitions as substitute for temporal leadership: An analysis of their effects on temporal conflict and team performance. *The Leadership Quarterly, 27*(4), 574-587. doi:https://doi.org/10.1016/j.leaqua.2015.12.002

Schaufeli, W. B., & Bakker, A. B. (2004). Job demands, job resources, and their relationship with burnout and engagement: a multi- sample study. *Journal of Organizational Behavior, 25*(3), 293-315. doi:10.1002/JOB.248

Shah, A. K., Mullainathan, S., & Shafir, E. (2012). Some Consequences of Having Too Little. *Science, 338*(6107), 682. doi:10.1126/science.1222426

Shakil, R. M., Memon, M. A., & Ting, H. (2021). Inclusive leadership and innovative work behaviour: the mediating role of job autonomy. *Quality & Quantity*. doi:10.1007/s11135-021-01102-0

Silva, A. (2021). Comparing teacher autonomy in different models of educational governance. *Nordic Journal of Studies in Educational Policy*, 1-16. doi:10.1080/20020317.2021.1965372

Soleas, E. K. (2020). Leader strategies for motivating innovation in individuals: a systematic review. *Journal of Innovation and Entrepreneurship, 9*(1), 9. doi:10.1186/s13731-020-00120-w

Stress, A. I. o. (2020). Stress, definition of stress, stressor, what is stress? Eustress.

Taylor, I. (2007). Discretion and Control in Education:The Teacher as Street-level Bureaucrat.

Educational Management Administration & Leadership, 35(4), 555-572. doi:10.1177/1741143207081063

Thurlings, M., Evers, A. T., & Vermeulen, M. (2015). Toward a Model of Explaining Teachers' Innovative Behavior: A Literature Review. *Review of Educational Research, 85*(3), 430-471. doi:10.3102/0034654314557949

Väänänen, A., & Toivanen, M. (2018). The challenge of tied autonomy for traditional work stress models. *Work & Stress, 32*(1), 1-5. doi:10.1080/02678373.2017.1415999

Väänänen, A., Toivanen, M., & Lallukka, T. (2020). Lost in Autonomy – Temporal Structures and Their Implications for Employees' Autonomy and Well-Being among Knowledge Workers. In (Vol. 4, pp. 83-101).

Vangrieken, K., Grosemans, I., Dochy, F., & Kyndt, E. (2017). Teacher autonomy and collaboration: A paradox? Conceptualising and measuring teachers' autonomy and collaborative attitude. *Teaching and Teacher Education, 67*, 302-315. doi:https://doi.org/10.1016/j.tate.2017.06.021

Xiao, H., Zhang, Z., & Zhang, L. (2020). Is temporal leadership always beneficial? The role of job passion and synchrony preference. *Personnel Review, ahead-of-print*(ahead-of-print). doi:10.1108/PR-02-2020-0078

Yidong, T., & Xinxin, L. (2013). How Ethical Leadership Influence Employees' Innovative Work Behavior: A Perspective of Intrinsic Motivation. *Journal of Business Ethics, 116*(2), 441-455.

Yuan, C.-C., & Lo, S.-H. (2018). Relationship Among Team Temporal Leadership, Competency, Followership, and Performance in Taiwanese Pharmaceutical Industry Leaders and Employees. *Journal of Career Development, 45*(3), 227-238. doi:10.1177/0894845316680087

Zhang, J., van Eerde, W., Gevers, J. M. P., & Zhu, W. (2021). How temporal leadership boosts employee innovative job performance. *European Journal of Innovation Management, 24*(1), 23-42. doi:10.1108/EJIM-05-2019-0112

Zhang, X., & Bartol, K. M. (2010). Linking Empowering Leadership and Employee Creativity: The Influence of Psychological Empowerment, Intrinsic Motivation, and Creative Process Engagement. *Academy of Management Journal, 53*(1), 107-128. doi:10.5465/AMJ.2010.48037118

Zhu, C., & Di, W. (2014). Key competencies and characteristics for innovative teaching among secondary school teachers: a mixed-methods research. *Asia Pacific Education Review, (ISI/SSCI) 15*, pp.299-311. doi:10.1007/s12564-014-9329-6

Zhu, C., & Engels, N. (2014). Organizational culture and instructional innovations in higher education: Perceptions and reactions of teachers and students. *Educational Management Administration & Leadership, 42*(1), 136-158. doi:10.1177/1741143213499253

Zhu, C., Wang, D., Cai, Y., & Engels, N. (2013). What core competencies are related to teachers' innovative teaching? *Asia-Pacific Journal of Teacher Education, 41*. doi:10.1080/1359866X.2012.753984

Fig 1. The mediation of job autonomy

Fig 2. The moderation of work stress

Fig 3. Simple slope analysis on the moderation of work stress

Fig 1. the mediation of job autonomy

Click here to access/download;Figure;figure1.tif

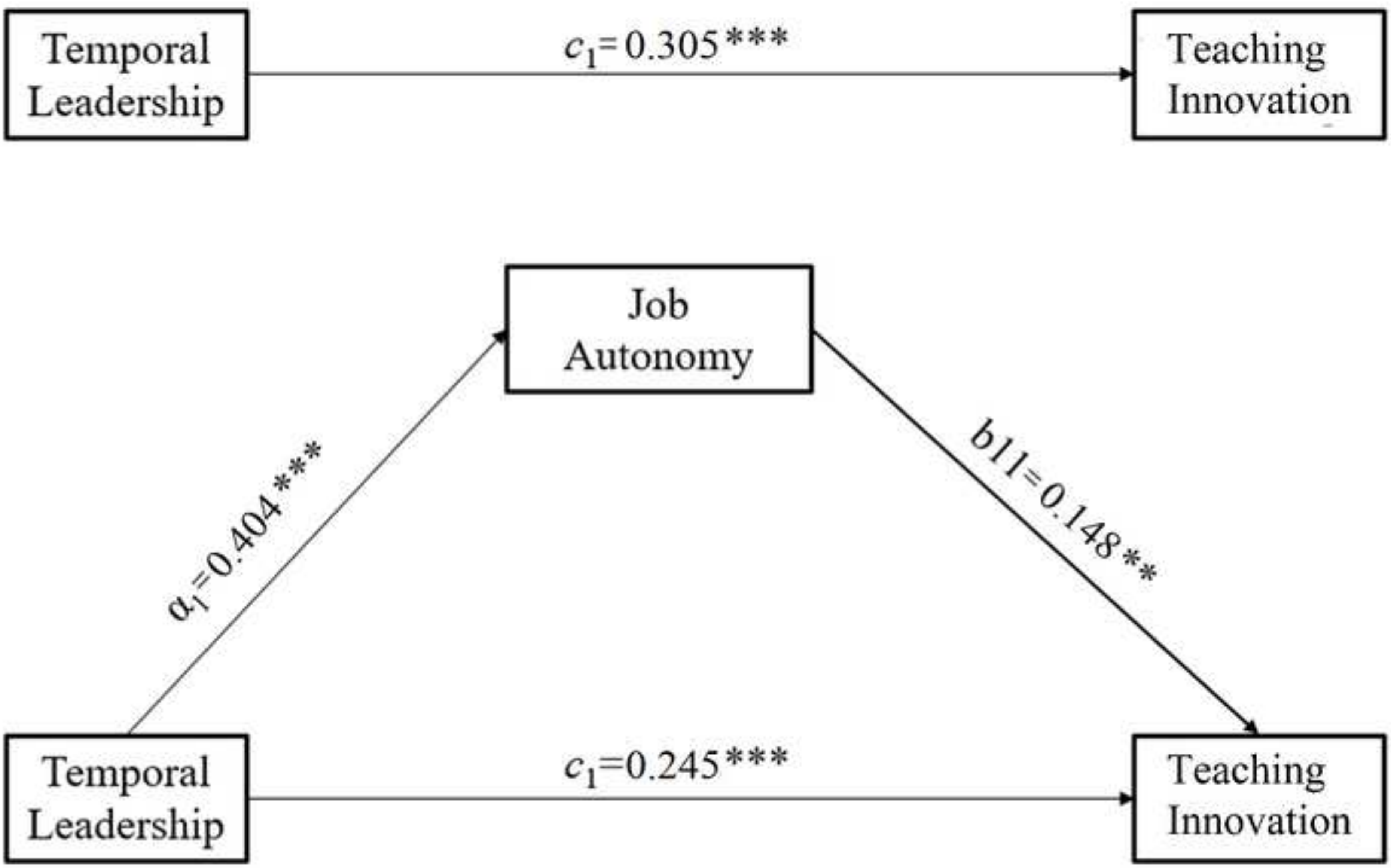

Fig 2. the moderation of work stress

Click here to access/download;Figure;figure2.tif

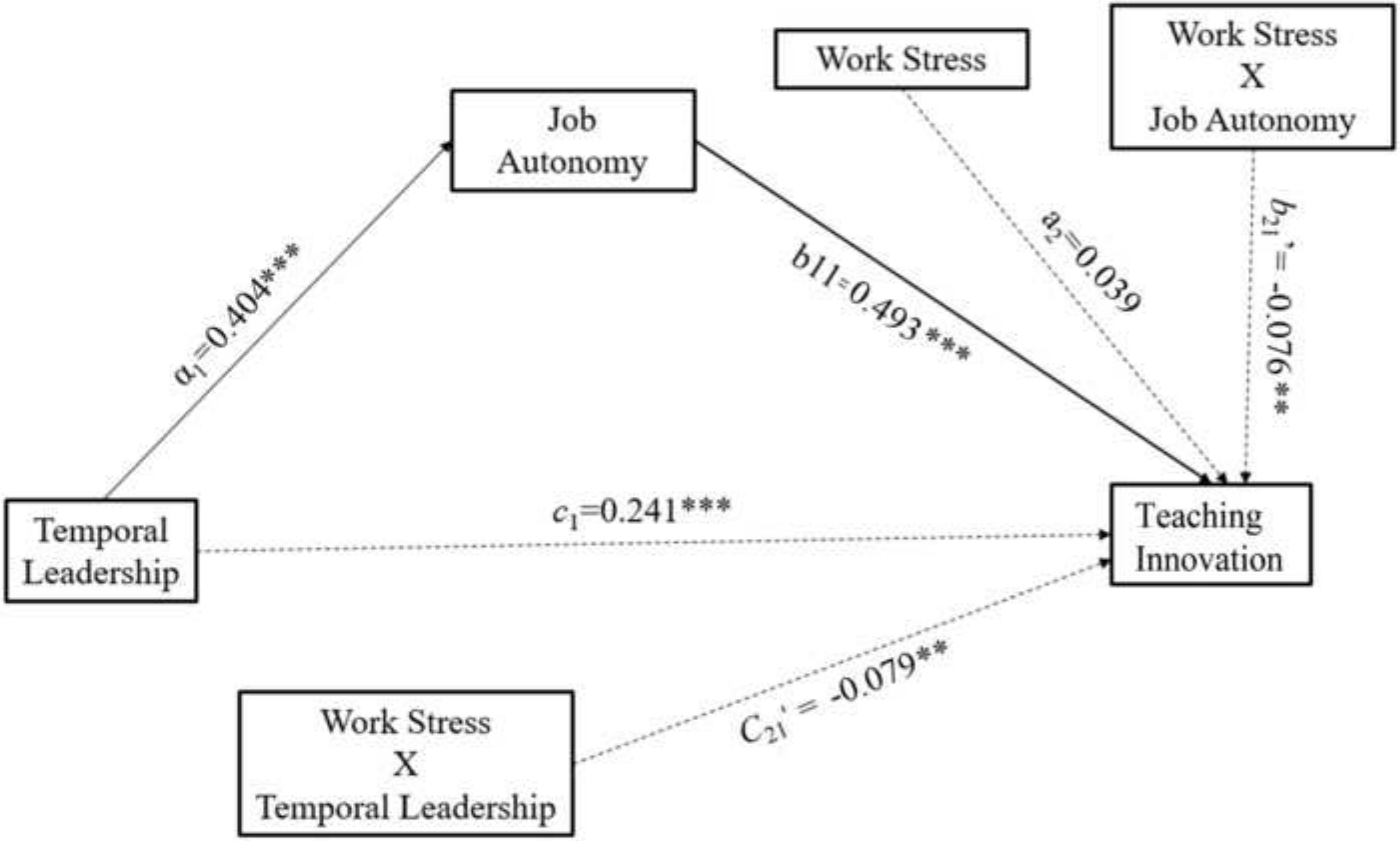

Fig 3. Simple slope analysis on the moderation of work stress

Click here to access/download;Figure;figure3.tif

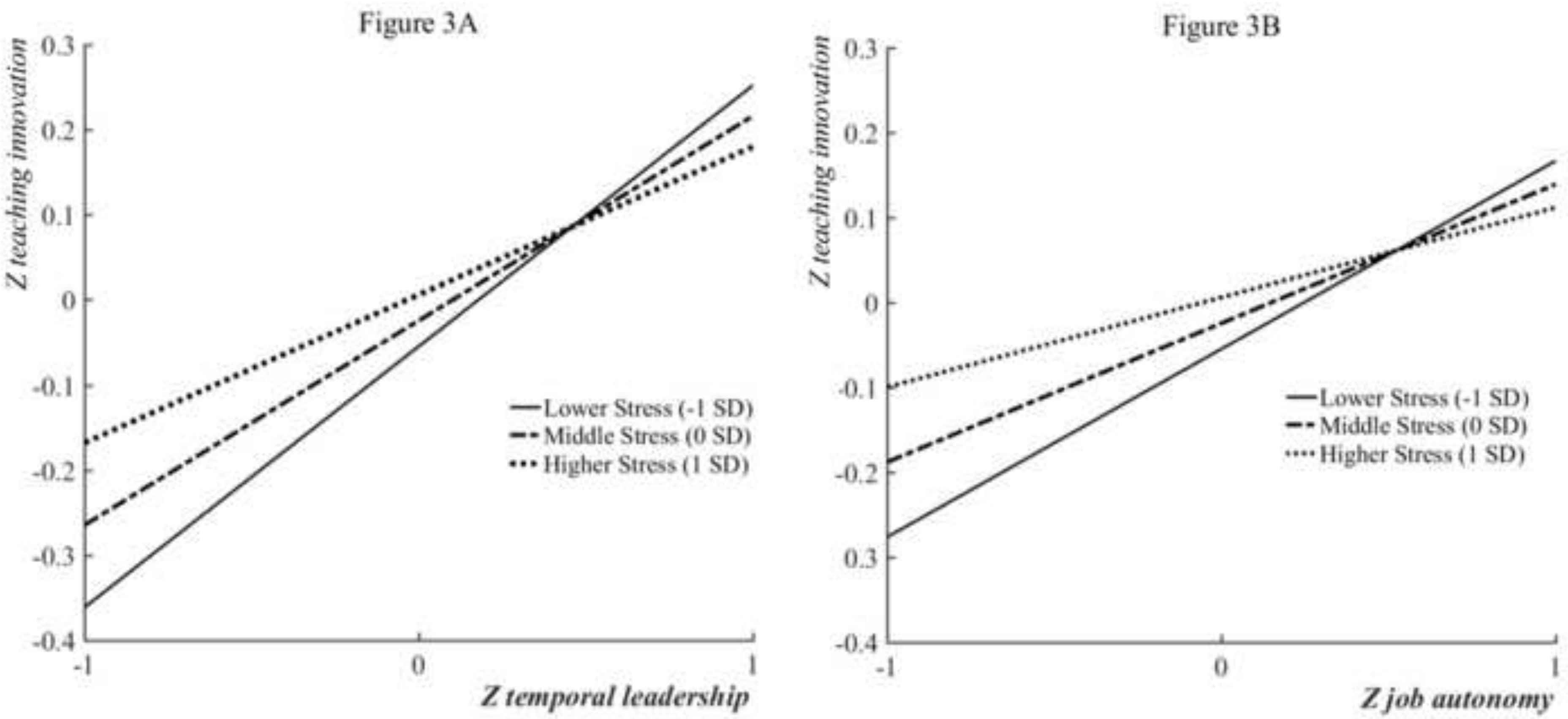

Supplement: Attachment — Submitted filename: PONE-D-22-11897_reviewer.pdf [file pone.0271195.s003.pdf]
