# Supplementary material for: Promoting teaching innovation of Chinese public-school teachers by team temporal leadership: The mediation of job autonomy and the moderation of work stress
Source: PLoS One. 2022 Jul 8;17(7):e0271195. doi: 10.1371/journal.pone.0271195 (PMC9269914; doi:10.1371/journal.pone.0271195)
Supplement: Attachment — Submitted filename: review_feedback_plosone20220612.docx [file pone.0271195.s004.docx]

Dear Editor(s) and reviewers:

We thank you, sincerely, for handling our manuscript and giving us valuable suggestions. We are encouraged by your positive feedbacks, including the novelty of research question, comprehensiveness of literature review, and importance of research findings, as well as the rigidity of the methodology highlighted by most of the reviewers.

We valued the suggestions deeply, and made accordingly one-to-one response to each of the concerns. We have integrated our replies into our revised manuscript, with changes highlighted at corresponding locations. We hope our revised manuscript has been substantially improved, and look forward to your good news.

Thank you very much.

Guiqin, Zhu

Journal Requirements:

When submitting your revision, we need you to address these additional requirements.

**1. Please ensure that your manuscript meets PLOS ONE's style requirements, including those for file naming. The PLOS ONE style templates can be found at**

**https://journals.plos.org/plosone/s/file?id=wjVg/PLOSOne_formatting_sample_main_body.pdf and**

[**https://journals.plos.org/plosone/s/file?id=ba62/PLOSOne_formatting_sample_title_authors_affiliations.pdf**](https://journals.plos.org/plosone/s/file?id=ba62/PLOSOne_formatting_sample_title_authors_affiliations.pdf)

We have read these two references and make changes to our manuscript accordingly.

**2. PLOS ONE does not copy edit accepted manuscripts (https://journals.plos.org/plosone/s/criteria-for-publication#loc-5). To that effect, please ensure that your submission is free of typos and grammatical errors.**

We have cross-checked the manuscript for typos and grammatical errors, we also invited one peer professor in English major to proofread the revised manuscript before submission.

**3. We note that the grant information you provided in the ‘Funding Information’ and ‘Financial Disclosure’ sections do not match.**

**When you resubmit, please ensure that you provide the correct grant numbers for the awards you received for your study in the ‘Funding Information’ section.**

We have checked the grant number and corrected it. The correct grant information is:

**Guiqin Zhu was supported by a grant from the ‘National Planning Office of Philosophy and Social Science, China’ (grant number: BHA18019). The funders had no role in study design, data collection and analysis, decision to publish, or preparation of the manuscript.**

Thank you for the message.

**4. Please remove your figures from within your manuscript file, leaving only the individual TIFF/EPS image files, uploaded separately. These will be automatically included in the reviewers’ PDF**

The figures have been removed in our revised manuscript.

**5. Please review your reference list to ensure that it is complete and correct. If you have cited papers that have been retracted, please include the rationale for doing so in the manuscript text, or remove these references and replace them with relevant current references. Any changes to the reference list should be mentioned in the rebuttal letter that accompanies your revised manuscript. If you need to cite a retracted article, indicate the article’s retracted status in the References list and also include a citation and full reference for the retraction notice.**

We have checked the references list, and replaced some old references (before 2010) with new ones (after 2010) as suggested by some reviewers. We have also changed the citation format into Vancouver as required by Plos One guideline. Details about the changes could be seen in our response to reviewer 2. As far as we know, no retracted references have been used in our article.

**Reviewer #1: This is quite a novel research area as there hasn't been many research on temporal leadership in education and teaching particularly. The literature review is quite comprehensive exploring important and key components of the research.
The findings of this research are an important contribution to literature on educational leadership and management, and temporal leadership and have important theoretical and practical implications. The application and pertinence of the paper are obvious, but the academic and theoretical value needs to be further improved through deeper research. Despite the comprehensiveness of the research, it may still be doubtful whether the findings of the study are generalizable or not.**

Thank you very much for your positive evaluation of our work. We agree with the reviewer over the limitations, especially the generalizability of our study. Actually, this is a concern shared by some other reviewers and we thank you sincerely for pointing it out and asking for further clarifications.

The findings in our study are based on subjective ratings from public elementary and secondary school teachers in mainland China. As we addressed in the ‘limitations’ section of our revised manuscript, given some unique characteristics in public school settings in the Chinese education system and the Eastern culture, there are a number of questions wide-open for future exploration, for instance, whether our findings could be generalized to private or charter schools, to education systems outside of mainland China. Additionally, because all the measurements in our study were self-reported, further studies, especially those based on objective measurements, could undoubtedly help us validate the effect of temporal leadership.

As our data collection has been finished and time and resources are quite limited for new round of surveys, we decided to elaborate these concerns by polishing our manuscript especially the ‘limitations’ section to, and suggesting further directions of research for exploration in the future. The ‘limitations’ section now reads (lines 595 – 622 on page 26):

*Despite the significance and strengths, the results of our study should be interpreted with caution. First, our study is based on observations of primary and secondary public-school teachers in mainland China. Chinese culture emphasizes social harmony, conformity, and collective thinking, which is quite different from the Western where individual contribution is more valued. There are also significant differences between countries in the systems of educational administration and pedagogy. For example, teachers in different countries vary in their understanding about teacher autonomy and pedagogical tasks (Silva, 2021). It is also worth noting that public demonstrate aspects of uniqueness from other institute types or organizations, such as private or charter schools where, according to some theories, competition is more prevalent and innovative behaviors are more emphasized. Thus, the question remains as to whether the same relationship between temporal leadership and job autonomy, temporal leadership and innovative behavior can be observed in other cultures, educational systems, and social sectors. Additionally, our study was based on teachers' self-assessment about teaching innovation and their supervisors’ team temporal leadership. Though subjective ratings could be positively associated with objective measurement (Koh & O’Higgins, 2018), it is still important to explore if our findings could be replicated with actual team temporal leadership and objective indices of teaching innovation. For instance, indices such as school performance (Kaiser, Hogan, & Craig, 2008) and contextualized behavior-based leadership assessment rubric (Koh & O’Higgins, 2018) can be referred for future studies. Finally, some studies have shown that innovation can be hindered when behavioral norms are strictly enforced or when lesson plans are far too detailed, for instance, micromanagement (Criscuolo, Salter, & Wal, 2014). Thus, the question arises as to what constitutes effective temporal leadership and what the difference is between the quality and the intensity of temporal leadership. It is also interesting to explore in depth the roles of discrete aspects of job demands and job autonomy (such as allowance for failure in innovation) or teachers’ self-efficacy. Future studies are needed to make these distinctions.*

Thank you for raising the concerns like generalizability. Despite these, we also hope our work could be shared by an internationally well-known platform given its significance and obvious strengths, as applauded by you and other reviewers. Since our paper is (at least among) the first to explore the issue of team temporal leadership in the realm of education, we are confident that it, if publicly available, could attract more researchers and give us encouragement for further exploration.

**Reviewer #2: I thank the authors for their efforts and will like to provide the following feedback for their consideration to enhance the paper:

Introduction
Some quotations need page number**

**Quotations need page number in in-text citations and more recent publications will add value to the paper**

Given that both questions asked for revision on citation format, we combined them and replied as follow:

Thanks for pointing this out. We have added page numbers to the following references due to short direct quotes, including ((Zhu & Di, 2014), page 300) in the first paragraph line 40 on page 3；((Bakker & Demerouti, 2014), page 9) in line 96 and line 99 on page 5.

We have also changed our citations into Vancouver format as required by the journal Plos One. The changes are made based on the tutorial website via <https://journals.plos.org/plosone/s/submission-guidelines> and the page numbers were added following <https://guides.library.uwa.edu.au/c.php?g=324981&p=2177205>. We welcome more detailed instructions from the editor or reviewers on this if further improvements are needed.

**Some references are old and need updating, e.g. 2003, 1979, 2011 and others**

Thanks for the suggestions about reference selection. In the revised manuscript, we replaced most the references before 2010 with more recent publications. The removed references include the following: Ancona et al., 2001; Halbesleben et al., 2003; Demerouti et al., 2001; Bakker et al., 2007; Schaufeli & Bakker, 2004; Goldsmith, 1986; Mishra & Koehler, 2006; Sagnak, 2012; Marks & Printy, 2003; Deci & Ryan, 2000; Bednall et al., 2018; Hallinger, 2003; Kirmeyer & Shirom, 1986; Fried & Ferris, 1987; Cribb & Gewirtz, 2007; Ferris, 1983; Kirmeyer & Shirom, 1986; Eagly et al., 2003; Breaugh, 1985.

Meanwhile, we decided to keep the following references because they are seminar articles conceptualizing the variables or proposing the theories of our research interests:

Karasek, 1979, which proposed for the first time the work demands and resources (JD-R) theory and work autonomy, and has 17352 citations according to google scholar up to May 30, 2022.

Hackman & Oldham, 1976 which proposed the job characteristics model and has 13890 citations up to May 30, 2022.

Ryan, Deci, 2000, which introduced the self-determinant theory and now has 49501 citations.

**In the abstract, 2021 surveys administered but 2017 indicated in the introduction. This needs to be fixed**

We are sorry for this confusion. We cross-checked our data and results outputs, and found that 2021 eligible teachers were included in our analysis. This error was then corrected in our revised manuscript, which can be seen in line 83 on page 4. Thank you very much for pointing out this mistake.

**Authors already proposed and concluded in the initial phase of the introduction before spelling out what they need to test as hypotheses.
Theoretical background and hypotheses?**

Thanks for this question. Since the article involves multiple variables of interests and lengthy reasoning of the hypotheses, we included an ‘introduction’ first section to address briefly the purposes and necessity of the research, and then the section ‘theoretical background and hypotheses’ to explain in details the key concepts, significant findings and main hypotheses in our research. The term ‘Theoretical background and hypotheses’ has been used in some publications in Plos One (Koob, Schröpfer, Coenen, Kus, & Schmidt, 2021; Miladinov, 2021; Moya-Clemente, Ribes-Giner, & Pantoja-Díaz, 2020; Pagliaro et al., 2021).

**Some hypotheses are tested and accepted. That is not enough to generalize. So, contextualizing this study can add value to the work**

We agreed with the reviewer for the limitations of our article particularly its generalizability. Actually, this concern is shared by some other reviewers, for instance, one reviewer asked the ‘difference between private and public schools’, and another one wondered if the findings could be generalized to education in other countries.

We thank the reviewer for the suggesting to contextualize the findings. Given these concerns and your advices, **we changed the title of our article into** ‘Promoting Teaching Innovation of Chinese Public School Teachers by Team Temporal Leadership: The Mediation of Job Autonomy and the moderation of Work Stress’.

**In our abstract, we changed our last sentence into**: These suggest that in our study context teaching innovation can benefit from leaders’ appropriate scheduling and synchronization of time resources, while emphasize the roles of job autonomy and work stress during this time-based team management.

**We also made noticeable changes and addressed these concerns in the ‘Limitations’ section.** Please see the changes from line 595 – 620 in page 26.

**Hypotheses 1 - 4 … in the study context? There is no need for splitting H4 into two.**

Thanks for your suggestion. We removed the content after the colon in H4 and now it reads as follow: *H4: Work stress moderates the effect of temporal leadership on teaching innovation*.

Please see the changes in lines 315-316 on page 14.

**3 Methods
Participants – Methods used and justifications? Justification of the sampling technique? Other ethical issues considered?**

We recruited our participants used a cluster sampling method, where 2021 valid samples were recruited from teachers in over 355 elementary or secondary public schools in mainland China. The survey was conducted in anonymity, and the participants were encouraged to respond to the questions but allowed to withdraw or skip the items they were reluctant to answer. We revised the ‘participants’ section by including more details about our data sampling as well as how the survey was conducted, which reads as follow (lines 319 – 337 on page 15):

*A total of 2021 valid samples were recruited from teachers in over 355 elementary or secondary public schools in 19 provinces in mainland China using a cluster sampling method. The response rate was 80.8%. This included 436 men and 1,585 women with a mean age of 36.67 years (SD = 8.92). 1% of them were with a diploma of high school or below, 27.1% with associate degree, 71.4% were with bachelor degree, and the other 0.5% were with master degree or higher. In term of work experience, 5.9% of them have taught for less than 1 year (1 inclusive), 18.3% for 1 to 5 years (1 inclusive), 19.8% for 5 to 10 years (10 inclusive), 25.3% for 10 to 20 years (20 inclusive), 24.3% for 20 to 30 years (30 inclusive), 6.1% for 30 to 40 years (40 inclusive). The number of participant teachers in these schools ranged from 1 to 53. In distributing the questionnaires, we treated the school unit as one group. First, the principal asked teachers to gather at a predetermined location. A trained research assistant then formulated the detailed guidelines, explained the implications of the survey, and ensured that anonymity was maintained. Teachers were asked to respond to all questions in the questionnaire according to their natural feelings. Important socio-demographic variables such as gender, age, and education level were also asked. The survey was conducted in anonymity, the participants were encouraged to respond to the questions but allowed to withdraw or skip the items they were reluctant to answer. The study was approved by the Ethic Committee of Xinyang Normal University (No. XYEC-2021-009), all the teachers gave written consents for participation.*

**Justifications for each approach described and data analysis strategies**

**No hypotheses have to do with demographics such as gender and age. Authors should decide on what they want to explore and take it forward**

**Results should focus on the findings. Strategies for minimizing biases to be transferred to the methods section**

Thanks for these three questions. Given all three questions are concerned with analysis strategies, we decided to combine them and reply in batch. Though age and gender exhibited nonsignificant relationships with the previous unidimensional measure of team temporal leadership (Mohammed & Nadkarni, 2011; Myer, 2010; Zhang, van Eerde, Gevers, & Zhu, 2021), previous studies have revealed gender differences in job autonomy (Petrie & Roman, 2004) and teacher innovation (Nguyen, Pietsch, & Gümüş, 2021), as well as age difference in job autonomy (Nguyen et al., 2021) and innovation (Nguyen et al., 2021). So, it is important to explore the relationship between our core variables of interests with gender and age under controlled. We therefore included them in the correlation analysis and then treated them as control variables followed previous studies (X. Liu, Baranchenko, An, Lin, & Ma, 2021; Nguyen et al., 2021).

We thank you again for asking us to clarify data analysis strategies. In the revised manuscript, we added the above information into our Method section, we also reallocated the strategies for bias minimization to the method section in the revised section. The section 4.1 now reads as follow (lines 401 – 404 page 18):

*Statistical analyzes were performed using IBM SPSS 20.0 software in the following steps: (1) descriptive statistics and correlation analysis for each variable; (2) common method bias analysis: to test the possibility of questionnaire bias (common method bias, CMB) (Podsakoff, MacKenzie, Lee, & Podsakoff, 2003), we anonymized all respondents and included all items of the above-mentioned 4 questionnaires and performed a principal component analysis. (3) regression analyses (the model 4) to assess the mediation of job autonomy on the effect of temporal leadership on teaching innovation; and (4) a conditional process analysis (model 15) was then performed to examine the moderation of job stress on the mediated effect of temporal leadership. In analysis 3 and 4, the number of the Bootstrap samples were set to 5000 with ‘Bias Corrected’ (Hayes & Rockwood, 2020). The confidence level for confidence intervals was 95% as default. Gender and age were treated as covariate variables in these two analyses followed previous studies (X. Liu et al., 2021; Nguyen et al., 2021). Though age and gender exhibited nonsignificant relationships with the previous unidimensional measure of team temporal leadership (Mohammed & Nadkarni, 2011; Myer, 2010; Zhang et al., 2021), previous studies have revealed gender differences in job autonomy (Petrie & Roman, 2004) and teacher innovation (Nguyen et al., 2021), as well as age difference in job autonomy (Nguyen et al., 2021) and innovation (Nguyen et al., 2021). So, it is important to explore the relationship between our core variables of interests with gender and age under controlled.*

**Discussion section should focus on the key findings related to the hypotheses along with the extant literature.**

**Implications should be reduced focusing on the critical consequences of the results.**

Thanks for the instructions. We have revised the discussion and implications sections at your request. For instance, we remove the first two sentences in the discussion section which seemed too-bold a claim. There are also many other changes which please see in our discussion and implications sections.

**Reviewer #3: I was unable to access the data provided. So I am assuming that the data were correctly computed and reported in the manuscript. Attached are my recommended edits for the authors line by line. In addition, all figures need to include footnotes re: level of statistical significance.**

We are sorry for this inconvenience and thank you for pointing out.

Originally, we have submitted our data in osf, and made it public via <https://osf.io/89xnh>;

Given that the data were not available at your convenience, we also uploaded the data to our github account, which can be reached unrestrainedly via the following address:

[*https://github.com/gqzhucqu/leadershipplosone*](https://github.com/gqzhucqu/leadershipplosone)

We also submitted the data as one part of the supplementary materials in our revised manuscript. The dataset file can be opened by SPSS.

We are especially grateful to your detailed editing suggestions and have made the changes as you suggested. As your question about the reference of Chen & Hu (2008), because this reference is a little old and another reviewer suggested us to replace it with new articles, we replace it with Wei et al. (2015) which proposed five factors for creative teaching. Please see the changes in lines 146-148 on page 7.

The footnotes about level of statistical significance have been added to the figures.

**Reviewer #4: Thank you for the opportunity to review your article, “Promoting Teaching Innovation by Team Temporal Leadership: The Mediation of Job Autonomy and the modulation of Work Stress.” This paper seeks an association between team temporal leadership and teaching innovation through the mechanism of job autonomy. Further, it explores the moderating effect of work stress on that association. This excellent paper shows an interesting and novel association between these constructs. In particular, the identification of job autonomy as a mediating variable is an fascinating and insightful contribution to the literature.

General: I am not aware of the term “modulation” when referring to an interacting variable. I have always read and used the term “moderation.” This may be a language translation issue. The authors should ensure that the term is being used properly and consistently throughout the paper.**

We thank the reviewer for the positive rating of the current article, as well as for pointing out this error. We regret for these translation inaccuracies and read Hay’s book (Hayes, 2018) to correct these errors. As you said, it should be ‘moderation’ instead of ‘modulation’. We have corrected these errors throughout the paper.

**2.2 The team temporal leadership of school principals may affect teacher innovative work behavior: As it is the focus of the paper, I would have appreciated a more thorough explanation of team temporal leadership.**

Thanks for this suggestion. In the revised manuscript, we added a direct quotation about the definition of ‘team temporal leadership’ at the very beginning of the section 2.2.1. This paragraph reads now as below, and please check our changes in lines 125 - 127 on page 6.

*Team temporal leadership (TTL) has been defined as “leader behaviors that aid in structuring, coordinating and managing the pacing of task accomplishment in a team” (Mohammed & Nadkarni, 2011, p.492). It concerns a unique dimension of leadership behavior that addresses time management in team activities (Mohammed & Nadkarni, 2011; Myer & Mohammed, 2012), such as scheduling, time synchronization, and time resource allocation.*

**2.4 The moderation of work stress: There should be citations for the assertions in lines 342-345.
I believe Hypothesis 4 is needlessly complex and could be ended at the colon. The mechanisms of how work stress moderates the association of team temporal leadership and teaching innovation are clearly explained in the Discussion.**

Thank you for these suggestions, and we replied to these two questions all together. In the revised manuscript, we have removed the content after the colon (lines 315 – 316 page 14), we have also changed some contents and added relevant references for introducing the moderation of work stress. The sections new read as follow (**note: in the manuscript the references were in Vancouver format, whereas in the current response letter we used the APA format to highlight the authors and publishing years for reviewers’ convenience**):

The literature has documented complex functions of work stress on organizational performance and innovation. *Behavioral and psychological models have identified two typical copying behaviors under stress: People revert to rigid and routine responses from previous experiences (Shah, Mullainathan, & Shafir, 2012) or make risky and erroneous decisions (Phillips-Wren & Adya, 2020). Stress is perceived by some researchers as an innovation killer (Cremer, Kalbe, Gläscher, & Schwabe, 2021; Plessow, Fischer, Kirschbaum, & Goschke, 2011). Under stress or when resources are scarce, people shift their attention to the problem at hand (Zhao & Tomm, 2018), reinforcing the urgency and importance of what is at hand at the expense of the future (Cannon, Goldsmith, & Roux, 2019; L. Liu, Feng, Chen, & Li, 2013; Shah et al., 2012). Stressful events may prompt reflexive or habitual reaction (Schwabe & Wolf, 2011), and impair the flexible adaptation to changing environment (Cremer et al., 2021). At the group level, some stressors such as time scarcity reduce team trust building (Bjorvatn & Wald, 2020), impair team knowledge transfer effectiveness (Bjorvatn & Wald, 2020).* However, there are also studies establishing positive effect of workspace stress on innovative work behavior (Daniel et al., 2020). Individuals under stress may be compelled to seek solutions and overcome challenges in an active problem-solving mode to self-develop and transform (Binnewies & Wörnlein, 2011). Under circumstances characterized by high job demands, stress could lead to higher levels of motivation, learning, and innovative behavior (Binnewies & Wörnlein, 2011; Schippers, West, & Dawson, 2015; Wang, Ren, Chadee, Liu, & Cai, 2021). We hence predict:

*H4: Work stress moderates the effect of temporal leadership on teaching innovation.*

**3. Methods: The primary thing I would like to see addressed in this paper is the distinction between administrators’ actual team temporal leadership vs. the participants’ perception of their team temporal leadership. I am concerned that teaching innovation is higher and work stress lower for teachers with higher self-efficacy, and those teachers may also be more likely to recognize and/or reap the benefits of team temporal leadership. Perhaps this could be controlled for if there are data on the participants’ sense of self-efficacy or other measures of teaching effectiveness.**

Thank you for these shrewd questions. The distinction between actual and perceived leadership is indeed an important question (Behrendt, Matz, & Göritz, 2017; Jacobsen & Bøgh Andersen, 2015; Koh & O’Higgins, 2018; Re & Rule, 2016). Given that there is discrepancy between self-rated and objective leadership performance, we strongly agree with the reviewer that the distinction between actual team temporal leadership (ATTL) and perceived team temporal leadership (PTTL) could contribute to the measurement and our understanding in the function of team leadership.

However, regrettably, till now almost all of empirical studies on temporal leadership were based on subjective rating by the team temporal leadership scale. Most of the studies asked the employees (Chen & Liu, 2020; Mohammed & Nadkarni, 2011; Myer, 2010; Santos, Passos, Uitdewilligen, & Nübold, 2016; Xiao, Zhang, & Zhang, 2020) to rate the temporal management of their supervisors, only quite few studies recruited managers as the raters (Chen & Liu, 2020). In other words, most of the existent studies are based on participants’ perception of the team temporal leadership. Since currently there has been no validated objective scale or matric for temporal leadership, our study also only used subjective ratings, with no objective or third-party measurements of temporal leadership behavior. Our study didn’t include any objective measurement of teaching innovation. Therefore, this limitation compromises the generalizability of our findings. In future studies, it would be mythologically important to develop valid objective metric for temporal leadership, and it would also be helpful to include certain objective measurements of teaching innovation. For instance, indices such as company profits which act as a “bottom-line” for businesses (Kaiser et al., 2008) and contextualized behavior-based leadership assessment rubric (Koh & O’Higgins, 2018) can be referred for future studies.

Despite these limitations, we would like to peddle, as a humble defense, that our study could be valuable for education management. On the one hand, representative leadership theories such as the leadership categorization theory (Lord, Foti, & De Vader, 1984) posits that common images of beliefs about appropriate leadership behavior and actions enable followers to use a consistent set of implicit beliefs to assess leaders. Empirical studies have found that perceived leadership could be positively associated with actual leadership measured by contextualized behavior-based leadership assessment rubric (Koh & O’Higgins, 2018), even children have implicit images or schemas of leaders (Antonakis & Dalgas, 2009). This suggested that, though with its innate weakness, peers’ or subordinates’ subjective rating could effectively reflect temporal leadership behaviors. Second, as our paper is (at least among) the first to bridge team temporal leadership and the realm of education, we hope its novelty and significance could attract ensuing investigation in temporal management issue in education and finally contribute to educational practices.

Meanwhile, regrettably our study did not include measurements about participants’ self-efficacy neither, so we cannot explore the role of self-efficacy in confounding the effect temporal leadership. But surely, it would be an interesting research question in our next study.

Given these considerations, we added these two weaknesses in the ‘limitations’ section of our revised manuscript. Please see the changes in lines 596 – 622 on page 27, or

**3.1 Participants: I would like to know how many different schools are represented in the sample.**
 Among the 2021 teachers included in our analysis, 1654 of them were from 367 schools based on the accurate logging of school names, the number of participant teachers varied between the minimum of 1 to the maximum of 53 across the 355 schools. Regrettably, for the remaining 367 teachers, their school names can’t be discerned due to technique failure (The survey was conducted in anonymity. The teachers were encouraged but not compelled to fill their affiliations. It turned out that part of them wrote down the names of their working town or village, instead of their school names. When analyzing the data, the authors and the assistants for data collection could not discern the affiliations these 367 teachers because there are more than one schools in the town/village involved). We have included this information in our revised manuscript, please see the changes 319 - 337 on page 15.

**3.2.4 Work stress: In lines 416-417, the sample question from the work stress questionnaire is not clear.**

Sorry for the typo in our previous manuscript, we have corrected this sentence which now reads as (lines 378 - 380):

*The teachers were asked to rate how stressed they felt in situations described by each item, for example: "the number of projects or tasks I have"*.

**5.1 Theoretical Implications: I am glad you made the assertion in lines 573-574 that “Temporal leadership is not synonymous with rigid time management that turns a blind eye to individual differences among team members.” Faced with the findings of this paper, disciples of Taylor’s Scientific Management might reflexively think that micro-management of staff’s schedule is an appropriate response. In fact, as you write, it is not.**

Thank you for raising this interesting question. A clear definition of team temporal leadership *(TTL)*, especially its uniqueness from other related concepts such as micromanagement, is core for our article. Some other reviewers have also required us to clarify this terminology, so in the revised manuscript, we first added a direct quotation of TTL by Mohammed & colleagues, which reads as follow: ‘*Team temporal leadership (TTL) has been defined as “leader behaviors that aid in structuring, coordinating and managing the pacing of task accomplishment in a team” (Mohammed & Nadkarni, 2011, p.492),* and change can be seen in Section 2.2.1 in our new manuscript.

Micromanagement is a management style where supervisors or managers closely observe and/or control the work of their subordinates or employees (Collins & Collins, 2002; White, 2010). Though it works in some circumstances (Collins & Collins, 2002), in majority of studies micromanagement is regarded negative and costly management style (Alvesson & Sveningsson, 2003; Collins & Collins, 2002; White, 2010). Given that temporal leadership ‘involves directive behaviors such as setting deadlines and urging members to complete tasks’ (Myer, 2010), it is natural to expect some correlation between temporal leadership and micromanagement, and it is also critical to distinguish these two concepts especially why temporal leadership was found beneficial as in our study.

To our best knowledge after searching for the references, currently there has been quite limited research addressing the association/distinctions between these two concepts. And here, we would like to refer to the articles by Myer (2008) and Adgate, Alipour (2020), the only two references we have found directly addressing these two concepts. As a pioneer who co-published the first questionnaire of team temporal leadership (Myer, 2010), Myer explained that micro-management (overmanaging) would be disruptive because it involved overcontrolling behaviors and taking away decisions from employees, which made subordinates untrusted and have less job autonomy (Myer, 2010). In contrast, as Myer said in his clarification, ‘the behaviors measured by the TOTL are indeed constructive rather than destructive and over-controlling’, and ‘While micro-management involves limiting subordinate’s participation, leaders high in ROTL tend to consult team members before making decisions about the use of time’ ((Myer, 2010), Page 53). Adgate, Alipour stated in their assay that ‘Keep in mind that this (temporal monitoring in temporal leadership) is not a form of micromanaging but a way of proactively gathering information’ ((Adgate & Alipour, 2020), page 5).

Myer also conducted questionnaire survey (Myer, 2010)) to estimate the correlation between temporal leadership and micromanagement, as well as their relationship with other management indices such as initiation of structure (example items: "assigns group members to particular tasks" and "makes sure that his/her part in the group is understood by the group members."), production emphasis (e.g. "permits the members to take it easy in their work" and "drives hard when there is a job to be done. "), consideration (e.g. "Does little things to make it pleasant to be a member of the group" and "puts suggestions made by the group into operation.”), liking (e.g. "I like my supervisor very much as a person” and "I think my supervisor would make a good friend.".

Myer’s study identified small but significant correlation between micromanagement and task-oriented temporal leadership (TOTL, r=0.22, p<0.01), but not significant correlation between micromanagement and relationship-oriented temporal leadership (ROTL, r=-0.05, p>0.05) ((Myer, 2010)), page 80). All three are positively associated with some leadership behaviors such as initiation of structure, production emphasis. **Importantly, temporal leadership demonstrated stark uniqueness in its correlation with other indices. For instances, both task- and relationship- oriented temporal leadership (TOTL and ROTL) are positively associated with consideration, transactional leadership, time management. ROTL is also positively associated with participants’ liking of their supervisors. In contrast, micromanagement was negatively correlated to all the latter indices. Additionally, micromanagement was found positively correlated to Laissez-Faire Leadership (LFL), but neither TOTL nor ROTL show significant correlation with LFL.**

Admittedly, though temporal leadership involves time monitoring, it can avoid time related conflicts and ensure that teams perform well (Santos et al., 2016). Managers with higher ability of temporal leadership can translate task temporal issues into clear guidelines for team members and develop an integrated and flexible approach for managing the team's temporal challenges and resources (Santos et al., 2016). In multiple studies, temporal leadership has been found beneficial to performance outcome (Maruping, Venkatesh, Thatcher, & Patel, 2015), such as innovative behaviors (Chen & Liu, 2020; Z. Liu, Liu, & Zhang, 2021; Xiao et al., 2020; Zhang et al., 2021). It is further found that higher temporal leadership can increase team performance amidst higher time urgency, but lower temporal leadership would decrease team performance under higher time urgency (Mohammed & Nadkarni, 2011). The positive effect of temporal leadership on innovation could be stronger when synchrony preference of employees (e.g. Sample items included “I am good at adapting to other people’s pace”) is higher (Xiao et al., 2020). So, according to these findings, leadership behaviors with temporal issues are not necessarily equal to tight-control as micromanagement, but in some scenarios, could be beneficial instead of destructive.

We applaud your professional sensitivity to the key terminologies in our article. Based on the above-reviewed evidence, we tentatively conclude that temporal leadership and micromanagement may be substantially different despite some superficial similarities. We also think that it would be interesting to include both of them in order to clarify the role of temporal leadership in future studies.

**Reviewer #5: Very interesting, well researched and well written study. Write-up is engaging to people interested in the different areas touched by this research such as leadership styles and facilitators for innovation take-up and implementation even if they are not working in the educational context. I have only included some minor editorial changes for the author's consideration.
Several interesting contributors were touched and discussed both at the literature review level as well during the discussion of the findings
The only major perspective that I missed seeing even a mention of is the effect of allowance for failure in innovation. Employees need to feel 'safe' that they will not be penalized should failure be the result of innovation. This perspective has also been researched extensively in its interaction with the take-up and implementation of innovation.**

Thank you very much for the positive evaluation and kind suggestions. Yes, failure is natural and essentially inescapable for innovation and scientific exploration (Dreysfuss, 2019). Despite being a necessary evil, failure often acts as a precondition for success. Individuals and organizations accumulate valuable experience by trial and errors before the final solutions come out (Townsend, 2010). From the perspective of organizational management, moreover, allowance for failure in innovation can create a supporting environment where the employees feel ‘safe’ as you mentioned and have the ‘job autonomy’ to navigate. This in turn increases morale and motivates innovation (Hutchison-Krupat & Chao, 2014; Manso, 2011). For instance, it has been found that IPO firms backed by more failure-tolerant venture capital investors are significantly more innovative and venture capital failure tolerance is particularly important for ventures that are subject to high failure risk (Tian & Wang, 2011).

We agree strongly with the reviewer that allowing for failure is pivotal to innovation, and it is worthwhile to be integrated into our manuscript. According to the Job-demand and resource theory (JD-R theory) on which our article anchors, allowing for failure in exploration could be regarded as a protecting job resource, one of ‘those physical, mental, social, or organizational aspects of work that are functional in (ⅰ) achieving work goals, and (ⅱ) promoting personal growth, learning, and development"’ ((Bakker & Demerouti, 2014), page 9). Given its significance and theoretical relevance, we therefore added it in several locations such as section 2.1 (lines 101 -102 on page 5) ‘*and typical examples of job resources are organizational support, job autonomy, and time control, allowance for failure (Hutchison-Krupat & Chao, 2014; Tian & Wang, 2011)*’.

We also included it in the section of ‘limitations’ by recommending it as an important research topic in following-up studies. Which could be seen in lines 596 – 622 on page 27, or on page 3 in the current response letter as our part of response to other reviewers.

Thanks very much for the suggestion.

**Reviewer #6: The study examines impact of temporal leadership on innovative teaching. This relationship is mediated via job autonomy for teaching which has been proven to improve teaching outcomes.

The following are my comments:

1. definition of temporal leadership can be more concise. Authors may cut down on extraneous details.**

Thanks for this suggestion. In the revised manuscript, we added a direct quotation about the definition of ‘team temporal leadership’ at the very beginning of the section 2.2.1. This paragraph reads now as below, and please check our changes in lines 125 – 127 on page 6.

*Team temporal leadership (TTL) has been defined as “leader behaviors that aid in structuring, coordinating and managing the pacing of task accomplishment in a team” (Mohammed & Nadkarni, 2011, p.492). It concerns a unique dimension of leadership behavior that addresses time management in team activities (Mohammed & Nadkarni, 2011; Myer & Mohammed, 2012), such as scheduling, time synchronization, and time resource allocation.*

**2. methods and results: the authors have done an excellent job in writing the study methods and explaining the results. It was easy to follow, I do not have any objections or concerns.**

Thanks very much for your appreciation.

**3. discussion: this line is not clear "515 Our findings reveal a clear message: we may not be able to schedule teaching
516 innovation, but we can schedule for it." Please reframe.**

Very good question. Actually, this concern was also raised by another reviewer. We totally agree with your opinion. Originally, we used this sentence to express the idea that ‘maybe we cannot schedule the progress of innovation, but we can make appropriate time arrangement or temporal leadership behavior to create an atmosphere which can benefit innovation’. However, as this sentence is vague, loosely-related to our findings, and a very bold claim, we decided to remove it and discussed our findings straightforward in the discussion section, which reads now as follows and please see the changes in lines 486 - 488 on page 22.

**Some typos:
line 47-- spacing between words
line 467--upper case "Job"**

Thanks for pointing out these typos. We have corrected them in the revised manuscript, please see the changes in lines 438. We have also cross-checked the spellings and grammar errors and invited an English professor to proofread the revised manuscript before submission.

**This is good work, can revise the above and resubmit. The manuscript is ready for publication in my opinion after addressing the above comments.**

Thank you very much again for your comments and suggestions.

**Reviewer #7: An important component of the sample should be described in more detail in the paper. Workplace autonomy and educational innovation have been shown to be stronger in schools where legislation on educational activities is more permissive. This is typically the case for private institutions, less so for public institutions. Therefore, it would be worthwhile to present in a few sentences that the sample includes responses from teachers in public or private schools. What is the proportion of responses received from teachers in private and public institutions? Is there a relevant difference between the answers received depending on whether the instructor giving the answer teaches in a public or private institution?**

We thank the reviewer for the comments, and totally agreed that it is worthwhile to include the public-versus-private school differences into consideration. This question is not only theoretically meaningful for revealing the boundary conditions for the effect of temporal leadership on teaching behaviors, but also helpful for education reforms and consumers’ school choice (Preston, Goldring, Berends, & Cannata, 2012).

It is natural to expect higher autonomy and educational innovation in certain types of institutes such as charter schools or private schools, and further, a unique relationship there between variables such as workplace autonomy and educational innovation. For instance, one theory holds that as private schools face more competition in consumer choice and resources, they are driven for diverse and innovative approaches (Walberg & Bast, 2003). Another theory proposes that private schools are free from bureaucracy, so educators there can have greater work autonomy, and greater opportunity to experiment with new strategies for improving achievement (Malloy & Wohlstetter, 2003). Echoing these theories, for example, a study from Turkey found that though private school teachers reported equal level of school climate for innovation in terms of support for innovation and resource allocation, they were self-reportedly more intrinsically motivated and creative than public school teachers (Fidan & Oztürk, 2015). Another study from Netherlands also found significant effect of school type on education performance which included innovation after some confounding variables were controlled (Haelermans & De Witte, 2012). There is also evidence that traditional public schools differ significantly from others like charter schools in terms of principal leadership style and leader turnover (Sun & Ni, 2016).

We also notice that there are some findings at odd with these hypotheses. For example, a study based on 2007–08 Schools and Staffing Survey (SASS) (NCES) in the United States (Preston et al., 2012). Similarly, a longitudinal study on American children found that Low-income children or children enrolled in urban schools probably benefited equally from attending private schools and public schools, because all of the advantages of private school education were eliminated when the sociodemographic characteristics that selected children and families into these schools got controlled (Pianta & Ansari, 2018). These mixed and unconclusive findings pinpoint to the necessity for further clarifications and boundary conditions about the effect of school system on educational variables (see a special issue, (Toma & Zimmer, 2012)), including our current study on team temporal leadership and teaching innovation.

However, regrettably, all the teachers in our study were recruited from public schools. So, we could not investigate the impact of school type on team temporal leadership as well as its relationship on teaching innovation, which may further challenge the generalizability of our findings. We therefore made multiple changes in our revised manuscript, including contextualizing our study in the article title, abstract, hypotheses and discussion sections. We stated explicitly that all our teachers were from public schools in the method section. Lastly, we revised the ‘limitations’ section by addressing the absence of private schools. Specifically:

The title now reads: ‘*Promoting Teaching Innovation of Chinese Public School Teachers by Team Temporal Leadership: The Mediation of Job Autonomy and the moderation of Work Stress*’

The abstract now reads: *This study examines the impact of team temporal leadership, leaders’ behaviors that help scheduling, allocating time resources, and coordinating team members, on teachers’ innovative behavior. Questionnaire surveys on 2021 Chinese elementary and secondary public-school teachers show that team temporal leadership exerts a significant positive direct effect on teaching innovation and the effect can be facilitated through the mediation of job autonomy. Moreover, both the direct effect and the second-leg of the mediation effect are moderated by work stress. These suggest that, at least in certain educational settings, teaching innovation can benefit from leaders’ appropriate scheduling and synchronization of time resources. The results also emphasize the roles of job autonomy and work stress during this time-based team management*.

The limitation section could be seen in lines 596 – 622 on page 27, or on page 3 in the current response letter as our part of response to other reviewers.

**References:**

Adgate, J., & Alipour, K. K. (2020). Temporal Leadership. Retrieved from <https://gradschool.ecu.edu/wp-content/pv-uploads/sites/118/2021/10/RCAW-Abstract-Book-2021.pdf>

Alvesson, M., & Sveningsson, S. (2003). Good Visions, Bad Micro-management and Ugly Ambiguity: Contradictions of (Non-)Leadership in a Knowledge-Intensive Organization. *Organization Studies, 24*(6), 961-988. doi:10.1177/0170840603024006007

Antonakis, J., & Dalgas, O. (2009). Predicting elections: child's play! *Science, 323*(5918), 1183. doi:10.1126/science.1167748

Bakker, A. B., & Demerouti, E. (2014). Job Demands–Resources Theory. In P. Y. Chen & C. L. Cooper (Eds.), *Work andWellbeing: Wellbeing: A Complete Reference Guide, Volume III* (pp. 1-28): John Wiley & Sons, Inc.

Behrendt, P., Matz, S., & Göritz, A. S. (2017). An integrative model of leadership behavior. *The Leadership Quarterly, 28*(1), 229-244. doi:<https://doi.org/10.1016/j.leaqua.2016.08.002>

Chen, J., & Liu, L. (2020). Reconciling temporal conflicts in innovation ambidexterity: the role of TMT temporal leadership. *Journal of Knowledge Management, 24*(8), 1899-1920. doi:10.1108/JKM-10-2019-0555

Collins, S. K., & Collins, K. S. (2002). Micromanagement--a costly management style. *Radiol Manage, 24*(6), 32-35.

Criscuolo, P., Salter, A., & Wal, A. L. J. T. (2014). Going Underground: Bootlegging and Individual Innovative Performance. *Organization Science, 25*(5), 1287-1305. doi:10.1287/orsc.2013.0856

Dreysfuss, E. (2019). Scientists Need to Talk More About Failure. Retrieved from <https://www.wired.com/story/scientists-need-more-failure-talk/>

Fidan, T., & Oztürk, I. (2015). The Relationship of the Creativity of Public and Private School Teachers to their Intrinsic Motivation and the School Climate for Innovation. *Procedia - Social and Behavioral Sciences, 195*, 905-914. doi:<https://doi.org/10.1016/j.sbspro.2015.06.370>

Haelermans, C., & De Witte, K. (2012). The role of innovations in secondary school performance – Evidence from a conditional efficiency model. *European Journal of Operational Research, 223*(2), 541-549. doi:<https://doi.org/10.1016/j.ejor.2012.06.030>

Hayes, A. F. (2018). *Introduction to mediation, moderation, and conditional process analysis: A regression-based approach (2nd ed.)*: New York, NY: Guilford Press.

Hayes, A. F., & Rockwood, N. J. (2020). Conditional Process Analysis: Concepts, Computation, and Advances in the Modeling of the Contingencies of Mechanisms. *American Behavioral Scientist, 64*(1), 19-54. doi:10.1177/0002764219859633

Hutchison-Krupat, J., & Chao, R. O. (2014). Tolerance for Failure and Incentives for Collaborative Innovation. *Production and Operations Management, 23*(8), 1265-1285. doi:<https://doi.org/10.1111/poms.12092>

Jacobsen, C. B., & Bøgh Andersen, L. (2015). Is leadership in the eye of the beholder? A study of intended and perceived leadership practices and organizational performance. *Public Administration Review, 75*(6), 829-841.

Kaiser, R. B., Hogan, R., & Craig, S. B. (2008). Leadership and the fate of organizations. *Am Psychol, 63*(2), 96-110. doi:10.1037/0003-066x.63.2.96

Koh, C. B., & O’Higgins, E. (2018). Relationships between emotional intelligence, perceived and actual leadership effectiveness in the military context. *Military Psychology, 30*(1), 27-42. doi:10.1080/08995605.2017.1419021

Koob, C., Schröpfer, K., Coenen, M., Kus, S., & Schmidt, N. (2021). Factors influencing study engagement during the COVID-19 pandemic: A cross-sectional study among health and social professions students. *PLoS One, 16*(7), e0255191. doi:10.1371/journal.pone.0255191

Liu, X., Baranchenko, Y., An, F., Lin, Z., & Ma, J. (2021). The impact of ethical leadership on employee creative deviance: the mediating role of job autonomy. *Leadership & Organization Development Journal, 42*(2), 219-232. doi:10.1108/LODJ-01-2020-0026

Liu, Z., Liu, X., & Zhang, X. (2021). How to Solve the Time Dilemma? The Influence of Team Temporal Leadership on Team Innovation Performance. *Frontiers in Psychology, 12*(3025). doi:10.3389/fpsyg.2021.634133

Lord, R. G., Foti, R. J., & De Vader, C. L. (1984). A test of leadership categorization theory: Internal structure, information processing, and leadership perceptions. *organizational behavior and human performance, 34*(3), 343-378. doi:<https://doi.org/10.1016/0030-5073(84)90043-6>

Manso, G. (2011). Motivating Innovation. *The Journal of Finance, 66*(5), 1823-1860. doi:<https://doi.org/10.1111/j.1540-6261.2011.01688.x>

Maruping, L. M., Venkatesh, V., Thatcher, S. M. B., & Patel, P. C. (2015). Folding Under Pressure or Rising to the Occasion? Perceived Time Pressure and the Moderating Role of Team Temporal Leadership. *Academy of Management Journal, 58*(5), 1313-1333. doi:10.5465/amj.2012.0468

Miladinov, G. (2021). The mechanism between mortality, population growth and ageing of the population in the European lower and upper middle income countries. *PLoS One, 16*(10), e0259169. doi:10.1371/journal.pone.0259169

Mohammed, S., & Nadkarni, S. (2011). Temporal Diversity and Team Performance: The Moderating Role of Team Temporal Leadership. *Academy of Management Journal, 54*(3), 489-508. doi:10.5465/amj.2011.61967991

Moya-Clemente, I., Ribes-Giner, G., & Pantoja-Díaz, O. (2020). Identifying environmental and economic development factors in sustainable entrepreneurship over time by partial least squares (PLS). *PLoS One, 15*(9), e0238462. doi:10.1371/journal.pone.0238462

Myer, A. T. (2010). *Team Temporal Leadership: Construct Development and Validation.* (Master Degree). The Pennsylvania State University,

Nguyen, D., Pietsch, M., & Gümüş, S. (2021). Collective teacher innovativeness in 48 countries: Effects of teacher autonomy, collaborative culture, and professional learning. *Teaching and Teacher Education, 106*, 103463. doi:<https://doi.org/10.1016/j.tate.2021.103463>

Pagliaro, S., Sacchi, S., Pacilli, M. G., Brambilla, M., Lionetti, F., Bettache, K., . . . Zubieta, E. (2021). Trust predicts COVID-19 prescribed and discretionary behavioral intentions in 23 countries. *PLoS One, 16*(3), e0248334. doi:10.1371/journal.pone.0248334

Petrie, M., & Roman, P. M. (2004). Race and gender differences in workplace autonomy: A research note. *Sociological Inquiry, 74*(4), 590-603.

Pianta, R. C., & Ansari, A. (2018). Does Attendance in Private Schools Predict Student Outcomes at Age 15? Evidence From a Longitudinal Study. *Educational Researcher, 47*, 419 - 434.

Podsakoff, P. M., MacKenzie, S. B., Lee, J. Y., & Podsakoff, N. P. (2003). Common method biases in behavioral research: a critical review of the literature and recommended remedies. *Journal of Applied Psychology, 88*(5), 879-903. doi:10.1037/0021-9010.88.5.879

Preston, C., Goldring, E., Berends, M., & Cannata, M. (2012). School innovation in district context: Comparing traditional public schools and charter schools. *Economics of Education Review, 31*(2), 318-330. doi:<https://doi.org/10.1016/j.econedurev.2011.07.016>

Re, D. E., & Rule, N. O. (2016). The big man has a big mouth: Mouth width correlates with perceived leadership ability and actual leadership performance. *Journal of Experimental Social Psychology, 63*, 86-93.

Santos, C. M., Passos, A. M., Uitdewilligen, S., & Nübold, A. (2016). Shared temporal cognitions as substitute for temporal leadership: An analysis of their effects on temporal conflict and team performance. *The Leadership Quarterly, 27*(4), 574-587. doi:<https://doi.org/10.1016/j.leaqua.2015.12.002>

Silva, A. (2021). Comparing teacher autonomy in different models of educational governance. *Nordic Journal of Studies in Educational Policy*, 1-16. doi:10.1080/20020317.2021.1965372

Sun, M., & Ni, Y. (2016). Work Environments and Labor Markets:Explaining Principal Turnover Gap Between Charter Schools and Traditional Public Schools. *Educational Administration Quarterly, 52*(1), 144-183. doi:10.1177/0013161x15616659

Tian, X., & Wang, T. Y. (2011). Tolerance for Failure and Corporate Innovation. *The Review of Financial Studies, 27*(1), 211-255. doi:10.1093/rfs/hhr130

Toma, E., & Zimmer, R. (2012). Two decades of charter schools: Expectations, reality, and the future. *Economics of Education Review, 31*(2), 209-212. doi:<https://doi.org/10.1016/j.econedurev.2011.10.001>

Townsend, W. (2010). Innovation and the Value of Failure. *International Journal of Management and Marketing Research, 3*(1), 75-84.

White, R. D. (2010). The Micromanagement Disease: Symptoms, Diagnosis, and Cure. *Public Personnel Management, 39*(1), 71-76. doi:10.1177/009102601003900105

Xiao, H., Zhang, Z., & Zhang, L. (2020). Is temporal leadership always beneficial? The role of job passion and synchrony preference. *Personnel Review, ahead-of-print*(ahead-of-print). doi:10.1108/PR-02-2020-0078

Zhang, J., van Eerde, W., Gevers, J. M. P., & Zhu, W. (2021). How temporal leadership boosts employee innovative job performance. *European Journal of Innovation Management, 24*(1), 23-42. doi:10.1108/EJIM-05-2019-0112

Zhu, C., & Di, W. (2014). Key competencies and characteristics for innovative teaching among secondary school teachers: a mixed-methods research. *Asia Pacific Education Review, (ISI/SSCI) 15*, pp.299-311. doi:10.1007/s12564-014-9329-6
